# Spirit of the Foreign Periodicals &c.

**Published:** 1840-01-01

**Authors:** 


### Spirit of the Foreign Periodicals.

### On the Duration of Human Life.

There is a very elaborate paper in a recent number of the Bulletin Belge on this subject by Dr. *Bellefroid,* who seems to have devoted much time and study to statistical researches. The following extracts will be read with interest.

*Duration of Generations.*

It is commonly believed that a generation lasts upon an average from 30 to 33 years, and thus that there are usually three generations, following each other, in the course of a century. The enquiries of M. *Villot,* the keeper of the Archives of France, confirm the truth of this opinion. From these it appears that the duration of a generation, in the *male* sex, is very nearly 33⅓ years among the upper classes of the Parisians, who have preserved their family records.

It is to be observed, however, that what M. *Villot* understands and means to denote by the expression *duration of male generations* is the interval lapsing between the birth of the father and that of his eldest son.

It would seem, therefore, that the duration of a generation—taking it in this sense—is very nearly the same as the medium or average duration of life in the country. In most of the countries of Europe, this may be estimated at 30 years, or thereabouts. Dr. *Caspar* of Berlin has with Germanic industry endeavoured to make calculations for the entire population of the world. He reckons the population at 960 millions, and the medium duration of life at 33 years: (this last number is certainly much too high.) From these data he concludes that upwards of 29 millions of human beings die annually, 80,000 daily, nearly 3,300 hourly, and 53 every minute. Well indeed may the learned Doctor exclaim, after announcing these awful ravages of insatiate Death, "What then are wars, and pestilences, and battles, and earthquakes, to the great household of creative nature!"

*Influence of Sex on the Duration of Life.*

The average life of males is in general shorter than that of females at every period of life. Even in reference to still-born children, there are many more such among male than among female births—omitting from our consideration the excess of the former over the latter.

In Belgium, of 2597 still-born children, 1517 were males, and only 1080 were females—a proportion of nearly 15 to 10, while the proportion of the male births to the females is as 15 to 14. The same result has been found at Hamburg, Paris, and Geneva; and we are told that in Prussia, during the years from 1826 to 1831, there were 59,144 still-born males, and only 43,533 still-born females. Dr. *Caspar* has calculated that, if the chances of life were equal in the two sexes, the number of still-born males should not have exceeded 45,709, instead of 59,144. It is certainly not easy to determine the cause of this very marked difference in the mortality of the two sexes at the period of birth. Dr. *Joseph Clarke,* and more recently Dr. *Lombard* of Geneva, have accounted for it by the greater size of the male over that of the female children,* and by the consequent greater impediments to an easy delivery.

But, although we admit this explanation, how are we to account for the female sex retaining the advantage at almost every period of life?

In England the probable or insurable life of female children, at the period of birth, exceeds by about five years that of males. It is true that they lose this advantage after the first year, and that their chances of life are even less than those of boys at 4, 7, 8, 9, 11, and 14 years of age; but at 15 years—the age which usually indicates puberty—they recover in part the advantage which they had at the period of birth, and they continue to retain it, with only slight exceptions, till about the 75th year, when the chances of the two sexes are about equal.

The following table will indicate the ratio of mortality among the two sexes, at different periods of life, in Belgium.

| AGE. | Loss per cent. among Males. | Loss per cent. among Females. |
|---|---|---|
| From 0 to 1 year | 22,0 | 17,6 |
| 1 — 5 years | 15,0 | 14,4 |
| 5 — 10 years | 4,5 | 4,1 |
| 10 — 20 years | 5,9 | 5,2 |
| 20 — 30 years | 7,5 | 8,8 |
| 30 — 40 years | 7,4 | 7,2 |
| 40 — 50 years | 5,7 | 6,7 |
| 50 — 90 years | 32,0 | 36,0 |

From this table it appears that, during the periods of fecundity and of change of life or catamenial cessation, the mortality is greater in the female than in the male sex; but, with these exceptions, the advantage is decidedly in favour of the *beau sexe;* and they scarcely deserve to be considered, if we bear in mind that the number of women, who reach the age of 20 years, is much greater than that of men.

It would seem, however, according to the results of M. *Quetelet's* enquiries, that there is a marked difference in reference to the advantage of female over male life in the inhabitants of the town and country. In the former, the advantage is decided and very considerable—nearly by *eight* years at the moment of birth,† and scarcely ever less than *three* years up to the 60th year; whereas in the latter it would appear, if the correctness of M. *Q.'s* statements be confirmed, that the chances are in favour of male life from the first to the fortieth year, and that it is only from this last named to the sixtieth year that female life has the advantage.

The following table of the mortality in a hundred among the two sexes will indicate the differences now mentioned, as observed in Belgium.

---

* The following table exhibits the average differences in point of weight and size of male and female children, according to the researches of M. *Quetelet.*

| | Weight | Size | Difference kilo. | Difference met. |
|---|---|---|---|---|
| Boys | 3 kil. 20 | 0 m. 496 | 0,29 | 0,13 |
| Girls | 2 kil. 91 | 0 m. 483 | | |

† It is supposed that in Geneva the probability of female life is superior to that of males by not less than twelve years, at the period of birth.

| Age. | In Towns. | | In the Country. | |
|---|---|---|---|---|
| | Males. | Females. | Males. | Females. |
| From 0 to 1 month... | 11,6 | 8,8 | 10,8 | 8,0 |
| 0 to 1 year..... | 25,8 | 20,7 | 24,3 | 20,0 |
| 1 to 5 years.... | 16,9 | 16,4 | 14,1 | 14,8 |
| 5 to 10 years.... | 3,5 | 3,8 | 4,3 | 4,4 |
| 10 to 20 years.... | 3,5 | 4,1 | 4,9 | 6,0 |
| 20 to 30 years.... | 7,0 | 6,7 | 6,7 | 6,7 |
| 30 to 40 years.... | 5,9 | 6,8 | 4,4 | 7,0 |
| 40 to 50 years.... | 6,5 | 6,1 | 5,5 | 6,6 |
| 50 to 90 years.... | 30,9 | 35,4 | 35,8 | 34,4 |

From this table it would seem that the country is favorable to the life of children of both sexes up to the fifth year, but that it is rather injurious from this date to the 20th year in males, and to the 50th year in females; for, whilst women of towns lose scarcely *one per cent.* more than men from the 30th to the 40th year—the chief period of fecundity—and the mortality among them remains even below that of the male sex during the period of the ferment of the passions—from the 20th to the 30th year—the loss during the former period among women of the country exceeds that of males nearly *three per cent.*, and during the latter period it is equal.

Whether this difference arises from the over-laborious life, to which the wives of peasants are subjected in Belgium, or from a greater mortality among them during accouchement in consequence of neglect and improper attendance, has not been ascertained.

Be this as it may, it seems now to be very generally admitted that *child-bearing does scarcely, if at all, add to the mortality in the female sex.*

This position seems to be established by comparing the results of reports from lying-in institutions with those of ordinary statistical tables. Of 118,187 women delivered in the Dublin Hospital from the year 1757 to 1828—and let it be remembered that a great many of these were destitute and sick when admitted—one only died in 83; and at Berlin, from 1819 to 1822, there was only one death in every 152 women who were confined; whereas, on an average in the same city, the mortality among men from 20 to 30 years of age is 1.3 in the same number 152. So much for the commonly supposed increase of mortality from child-bearing.

Let us now see whether there are any real grounds of alarm for what has been called the *critical epoch of female life.* According to the researches of M. *Benoiston* at Paris, Berlin, Petersburg, and in Sweden and Switzerland, it appears that the mortality in a hundred among each sex between the ages of 40 and 60 years of life is as follows.

| Age. | Men. | Women. |
|---|---|---|
| 40 | 8,2 | 7,7 |
| 45 | 10,3 | 8,7 |
| 50 | 12,6 | 9,6 |
| 55 | 14,7 | 11,1 |
| 60 | 17,7 | 13,6 |

So that, if there is in the human race any thing *critical* between the ages of 40 and 55 years of life, it is rather in the male than in the female sex. We may therefore conclude with M. *Benoiston,* that "if we examine the mortality among the female sex from the 40th to the 60th degree of north latitude on a line extending from Marseilles to Petersburgh, and passing by Nevers, Paris, and Stockholm, we shall not find, at any epoch of life from the 30th to the 60th year, a sensible augmentation (of the mortality), beyond what may be expected from the natural effect of the advance of age; whereas, at all the epochs of life in males from their 30th to their 90th year, we observe a considerably greater increase of mortality among men than among women."

It would appear therefore, that there is no real foundation for the common idea that the critical (so called) period of life among the latter affects the rate of mortality in the sex.

It is certainly not very easy to account for the almost uniform greater mortality among males after the period of puberty. Writers have alluded to the destruction of health from the dangers and excesses, both physical and moral, to which the one sex is exposed more than the other, to the havocs of war, to the losses at sea, &c.; but these influences, although certainly not inoperative, are not sufficient to explain the question at issue. M. *Benoiston* has found that a similar excess of mortality in the one sex exists among monks and other male recluses, compared with that among nuns, as is indicated in the following table:—

| AGE. | Mortality per cent. among Monks. | Mortality per cent. among Nuns. |
|---|---|---|
| 30 | 8,3 | 7,9 |
| 40 | 9,5 | 9,2 |
| 50 | 12,8 | 11,5 |
| 60 | 18,2 | 15,0 |
| 70 | 22,6 | 21,5 |
| 80 | 21,3 | 22,8 |
| 90 | 7,3 | 11,1 |

(The results of this table, it will be noticed, corroborate the previous remarks on the rate of mortality at the *critical* period of life in the two sexes.)

The most puzzling part of the whole problem is to account for the greater mortality in the male sex during the first year of life—and, we may add, before birth, as indicated by the number of still-born children—before any of the physical or moral influences, which have been often considered as affording a solution of the difficulty, are brought into play.

We shall now proceed to another topic of this statistical enquiry,

*The Influence of Marriage and of Fecundity on the Duration of Life.*

*Hufeland* and *Deparcieux* were the first who affirmed that celibacy shortens life. Dr. *Odier* concludes, from the results of very numerous enquiries and calculations, that the average or medium life of a married woman is always superior to that of an unmarried one, except perhaps in extreme old age; at the age of 20, the difference is at least 10 years in favour of the former. The correctness of this conclusion has been amply confirmed by the researches of M. *Caspar* of Berlin, and also of M. *Benoiston.* The former of these authors gives the following table, prepared from the mortality registers of Amsterdam from the year 1814 to 1826.

MORTALITY IN A HUNDRED.

| AGE. | Husbands or widowers. | Unmarried males. | Wives or widows. | Unmarried females. |
|---|---|---|---|---|
| From 20 to 30 years | 3,6 | 43,1 | 4,7 | 26,5 |
| — 30 to 45 — | 17,9 | 27,1 | 16,5 | 24,5 |
| — 45 to 60 — | 29,2 | 15,6 | 22,6 | 19,2 |
| — 60 to 70 — | 22,0 | 8,1 | 22,3 | 13,0 |
| — 70 to 80 — | 19,4 | 4,3 | 22,9 | 11,6 |
| — 80 to 90 — | 7,0 | 1,4 | 9,6 | 4,1 |
| — 90 to 100 — | 0,8 | 0,0 | 1,2 | 0,7 |

It should be remarked, in reference to the difference of mortality among the married and unmarried men from the age of 20 to that of 30 years, that the disproportion is not really so great as the table seems at first sight to indicate; for in Holland, as in Belgium, the greater number of marriages among men occur at from 28 to 30 years of age; and those persons who marry earlier are generally in easy circumstances—the favourable influences of which on the duration of life we shall presently shew. On the other hand, the apparent excess of mortality among married men, as life advances, will deceive us, unless we keep in mind that at the age—say of 45 years—a much greater number survive among them than among *celibitaires.* It is however remarkable that of an equal number of 100 individuals of each class there survive 27,2 married men at 70 years of age, whereas there remain only 5,7 celibitaires at the same period of life. M. *Benoiston* is of opinion that the *probable* life of a married man at the age of 20 exceeds by 19 years that of a bachelor at the same period of life.

The beneficial influence of marriage on the duration of life is certainly more striking in the male than in the female sex; but even in the case of the latter, it is only necessary to look over Caspar's table to discover that at almost every period of life, even during the periods of fecundity and of the critical change, the advantage is decidedly in favour of married women.

M. *Benoiston* has calculated what he considers to be the chances in favour of married over unmarried men at different periods of life, and the results may be briefly stated in the following table.

| Age. | Difference in favour of married men. |
|---|---|
| 30 | 17 years. |
| 45 | 18 years. |
| 60 | 8 years. |
| 70 | 4 years. |
| 80 | 1 year. |
| 90 | 0 year. |

(In conclusion, we can assure the curious reader that he will find the original paper by M. *Bellefroid* very interesting and worthy of study. It is in the last August number of the *Bulletin Medicale Belge.)*

It may be useful as well as interesting, to subjoin to the preceding paper the following table, shewing the proportion of deaths out of 10,000 deaths in males and females, according to the London Registers of Burials 1813—1830, and according to the Registers of Deaths for the year ending June 30, 1838.

[This table appeared in the last number of this Review, p. 445.—*Rev.*]

| Age. | Registers of Burials. | | Registers of Deaths. | |
|---|---|---|---|---|
| | Males. | Females. | Males. | Females. |
| Under 1 year ........ | 2,188 | 1,756 | 2,339 | 1,933 |
| From 1 to 5 years ... | 1,498 | 1,450 | 1,742 | 1,780 |
| 5 to 10 years.... | 437 | 410 | 457 | 462 |
| 10 to 20 years.... | 579 | 636 | 554 | 647 |
| 20 to 30 years.... | 724 | 839 | 738 | 833 |
| 30 to 40 years.... | 621 | 725 | 655 | 715 |
| 40 to 50 years.... | 649 | 670 | 653 | 632 |
| 50 to 60 years.... | 715 | 784 | 656 | 621 |
| 60 to 70 years.... | 911 | 922 | 813 | 823 |
| 70 to 80 years.... | 1,012 | 1,086 | 830 | 883 |
| 80 to 90 years.... | 584 | 700 | 473 | 556 |
| 90 and upwards.. | 75 | 116 | 94 | 115 |

### ON DRUNKENNESS AMONG THE MANUFACTURING CLASSES IN FRANCE.

A report upon the physical and moral condition of the working classes in France was recently submitted to the attention of the Institute by M. *Villermé*, who has long devoted much of his time and attention to all subjects connected with the jurisprudence of medicine. The following extract on the pernicious influence of that most slavish of vices, drunkenness, will shew how widely and banefully spread it is among the manufacturing population in that country.

" It would seem that high wages are seldom or ever any great boon to the workman; for the more they gain, the more they will spend; so that, at the close of the year, there is rarely any difference in the means or property of the best and of the worst-paid among them.

At the same time it deserves notice that, although they usually consume a large quantity of food when they can procure it, they know how in times of distress to bear the greatest privations. The worst privation to bear, at least to many of them, is the want of wine and other stimulating liquors. It is unfortunately too much the case that, just in proportion to the poverty and distress of the workmen, so is their desire for such intoxicating drinks—no doubt because these make them forget their misery for the time.

Among the working as among the other classes of the lower orders, drunkenness is almost exclusively found among the men; and with them, it has been remarked, that the pernicious practice is seldom acquired before their first illicit connexions with women. It is most common among those who are either exposed much to heat, or have severe muscular exertion during their occupations; and it is more common in the cold climate of the northern than in the warmer climate of the southern provinces. In Lille perhaps more than in any other town the vice is most frequent and most revolting.*

* In the northern countries of Europe the spirit, that is commonly used, is obtained either from barley or from potatoes. The cheapness of the latter kind, and the great number of distilleries which prepare it, have contributed not a little to the increase of drunkenness.

The causes, which most contribute to engender and increase the disposition to drink intoxicating liquors, are

1. The bad examples which the youth from their very infancy see before them.

2. The choice of apprenticeship to a trade in which many drunkards are employed.

3. The habits of debauch and disorderly life induced by frequenting clubs and other social meetings.

4. The complete idleness on Sundays and on other holidays.

5. The low price of brandy and other spirituous liquors, and the number of cafés and taverns everywhere.

6. The destitution of moral and religious feelings and practices.

The practice of drinking is unquestionably at first acquired by mere imitation, and by a silly desire to be able to do as others do; then a pleasant sensation takes the place of indifference or of even a positive disrelish, until at length an irresistible desire and increasing passion enslave the degraded victim. It is thus that, step by step, and often alas! by a rapid transition, men pass from a decent sobriety to a most dreadful intemperance. When once the habit is acquired, every thing becomes, so to speak, an occasion to visit the *cabaret*. When work is brisk and he receives good wages, he goes because he has plenty of money; when out of employment, he goes because he has nothing else to do; when happy, to enjoy himself; and when unhappy, to forget his distresses. It is at the *cabaret* that he incurs his debts, that he pays them when he can, that he concludes his bargains, and contracts his acquaintanceships; in short, every thing is done at the *cabaret*, until the unhappy man is utterly ruined or is driven to destroy his own life.

When it has reached this pass, not only is all thrift, attention to children, and domestic happiness at an end, but the infatuated man and his family are brought to beggary and want; he is now idle, quarrelsome and discontented, fond of gambling and low play; he becomes degraded in his own estimation, and revengeful against others more fortunate or less vicious than himself; his health is impaired, and his feelings are brutalized; he associates with vice till he forgets that it is so; and at length he gives himself up to the perpetration of some crime which makes him an inmate of a jail.

It may be safely affirmed that drunkenness is the chief cause of almost all the disturbances and insurrections among our working classes, and of the greater number of crimes and offences against the laws.

The greatest blessing that could be conferred upon them would be, not to give them high wages, not to provide them with pleasures and entertainments, but simply to introduce habits of sobriety among them.

But how is this to be effected? Some will answer, by bringing the workman to make it a point of honour to himself to give up the practice, and by inducing him to become a member of a temperance society. The dissemination of books and pamphlets, whose object is to point out the numerous mischiefs arising from intoxication, the quarrels it gives rise to, the vices and crimes of which it is the parent, the ruin of health and strength and the debasement of mind which it occasions, should certainly be encouraged not only by the efforts of private charity, but by the direct interposition of all enlightened governments; while others suggest the necessity of restrictive duties upon all distilled spirituous liquors, and of severe police regulations against public-houses being open at late or very early hours. Good may be expected from adopting both of these plans—by appealing to the better feelings of the poor, and at the same time by discouraging and preventing, as far as possible, the sale of ardent spirits for drink by means of high fiscal duties.*

---

* Every one residing in London—and we suppose it is, or will soon be, the

M. *Villermé* had consulted several of the old workmen, who had become masters themselves, as to the most advisable means to prevent and discourage the habits of drinking among the manufacturing classes. Their suggestion was, that, for the first offence, the offender should be reprimanded by a magistrate, or even be imprisoned for a few hours, or for a day at most; and that, if the offence were repeated within a given time, the name of the culprit should be published to the neighbourhood. They believed by thus appealing to the honorable feelings of young men, and at the same time by holding out the threat of public exposure, a great restraint might be imposed upon not a few, at least.

To effect the cure of the wide-spread evil of intoxication, religion as well as municipal authority, the clergyman and the magistrate, the gentry and the master-manufacturers should all combine together in a spirit of active co-operation and unanimity. Much might certainly be done, if the *chefs de fabrique* —in other words, the proprietors of manufacturing establishments—were animated by a more philanthropic and christian spirit. Unfortunately, every thing is sacrificed to the ambition of mere wealth.

There are however honorable exceptions occasionally to be met with; and they well deserve to be noticed. M. *Villermé* particularly mentions the praiseworthy conduct of the leading manufacturers at Sèdan, who by discouraging the keeping of holidays and of not working on Mondays, by retaining in their employ such workmen as fall sick, and treating them all with kindness but at the same time with firm discipline, and by discharging any, never to receive them back, who are seen drunk, have contributed a great deal to improve the general character of the lower classes in the town.

We do not wish any one to imagine that we are opposed to the workman taking a single glass of wine, or beer, or of cyder: we rather wish that every man could have a moderate allowance of any of these liquors at his meals; the evil is not so much in the act as in the abuse and excess of it. It is however to be remarked that, according to the reports of the American Temperance Societies, no progress will be made in eradicating the evil of drunkenness, but by inducing the members to abstain from spirituous liquors *altogether*, and not merely by limiting the use to a certain quantity, or by taking them in moderation. The extraordinary success which, we are told, has attended the exertions of some of these societies, cannot but delight every well-thinking mind.

It is a common—but, we believe, a mistaken notion—that it is easier for a person to moderate the quantity of an accustomed stimulus, than to forego its use altogether. Reformed drunkards seem to agree that such is not the case; the entire withdrawal is easier to bear than the limited allowance.

The position therefore of the American Temperance Societies is founded on just grounds—*that a complete abstinence from intoxicating liquors is the only certain remedy against intemperance.*

It would seem that hitherto temperance societies have made but little ground in France, and M. *Villermé* appears not to anticipate nearly so much good among his countrymen from them as from well-organized associations of the leading manufacturers for the purpose of encouraging and exacting habits of sobriety from the workmen in their employment. He adds; *I consider the religious spirit of the United States as a powerful element in the success of temperance societies, which does not exist in our population.—Annales d'Hygiene Publique.*

---

same in all towns in England—must have been gratified by the change in the appearance of the streets on Sunday mornings, since the New Police Bill came into effect about three or four months ago. No public-house is allowed to be open between twelve o'clock on Saturday night, and one o'clock P. M. on Sundays.—(*Rev.*)

### Use of the Microscope in Legal Medicine—Detection of the Spermatic Animalculæ.

The French physicians have, during the last few years, been prosecuting with their accustomed zeal various subjects of physiology by means of microscopical enquiry. There cannot be a doubt that the value of the microscope has perhaps never, and certainly not during the last half century, been appreciated by the medical profession as it well deserves to be.

The following extracts from an elaborate paper by Dr. *Bayard,* on the *microscopical examination of seminal or spermatic stains on linen and other articles of clothing,* may suffice to shew the assistance to be derived from its adoption. The employment of the microscope, says he, in medico-legal experiments was first practised and recommended by M. *Orfila,* to ascertain the presence and nature of the fluid within the vagina and on the external parts, whether spermatic or not, in cases of suspected rape. His enquiries did not however, it would seem, lead to any satisfactory results; as he distinctly stated that, " no conclusion can be drawn from microscopical examinations in determining the existence of spermatic spots or stains."

It is to M. *Ollivier* that we are indebted for the first conclusive application of the microscope in medico-legal experiments. In June 1837, he was appointed to determine *if there was any hair or portion of hair adhering to a hatchet found in the house of a person accused of murder, and, if so, to say whether these hairs were human or not.* After a very careful examination, M. O. deposed that there were certainly portions of hair sticking to the blade of the instrument, but that it was not human hair, but that of some of the lower animals.* The correctness of the opinion was confirmed by the result of the judicial enquiry.

M. *Ollivier* has since published, in the Archives de Medecine for December 1838, the account of a novel application of the microscope, to ascertain the purity and genuineness of opium.

In November of the same year M. *Devergie* read before the Academy of Medicine a note on the characters or signs of death from hanging, and he particularly insisted upon the presence of spermatic animalculæ in the male urethra, and of the general congestion of the generative organs, as among the most important of all.†

We must not omit to mention that M. *Donné* had availed himself of the microscope in studying the characters of some animal secretions: his two memoirs "On the Nature of the Mucus and the Matter of Discharges from the Genital Organs in Men and Women, and also of the Spermatic Animalculæ" were published during the course of 1837.

In the latter of these papers, he endeavoured to point out the circumstances that were favourable and unfavourahle to the life of these animalculæ, and he deduced certain conclusions as to some of the causes of sterility in women.‡

Dr. *Bayard* has, within the last year or two, followed out the suggestions of M. *Orfila* as to the evidence which might be derived from the microscopical examination of spermatic stains in certain medico-legal enquiries, and he has been enabled to correct the erroneous and unfavourable conclusion to which this distinguished physician had arrived.

He shews that it was in consequence of rubbing or beating in the water the linen on which were the spermatic stains, that the animalculæ were so bruised and broken in pieces that they could no longer be distinguished by any micros-

---

* Vide Medico-Chirurgical Review, July 1839.
† Vide Medico-Chirurgical Review, October 1839.
‡ Vide Medico-Chirurgical Review, April 1838.

cope however powerful. By simply moistening the linen in water, contained in a watch-glass, for several hours and then heating it gently with a spirit-lamp, he succeeded in detecting the dead animalculæ without any difficulty. Along with these animalculæ may usually be observed some prostatic *monades*: these have a globular form and are without tails, and they are considerably larger than the genuine *zoospermes* or spermatic animalculæ, which, it is well known, have tails, and very much resemble in shape miniature tadpoles. The most important caution in conducting the above experiment is to avoid in any way bruising the animalculæ by rubbing or squeezing the linen. When this has been macerated for a sufficient length of time, Dr. B. sometimes filters the fluid, and thus procures all the solid matter on the surface of the filtering paper. He then takes a small portion of this and drops a little alcoholised or ammoniated water upon it for the purpose of dissolving its mucous portion, and of detaching the zoospermes from its surface: if any fatty matter is present, a few drops of etherialized water should be used. At the bottom of the fluid thus obtained, a deposit will quickly be perceived: of this a few drops may be sucked up by means of a small tube and placed on the object-glass of a microscope which magnifies 400 or 600 times.

When thus examined, we usually perceive between the plates of glass several spots of a greasy aspect; it is in these spots that the animalculæ are generally observed. In other points we may notice numerous corpuscles suspended in the fluid, and perhaps also several of the animalculæ floating freely about.

There is another method that may be followed. A few drops of the fluid may be placed on a portion of glass and allowed to become dry; if it is now subjected to examination with the microscope, the zoospermes will be generally very easily detected.

Dr. *Bayard*, after giving a most minute account of the numerous experiments* which he has performed, sums up the results of his observations in the following category of conclusions:

1. The spermatic animalculæ retain their life and movements, as long as the mucus, in which they are suspended, remains fluid and warm. I have found them alive during ten hours.

2. The semen, if allowed to become dry and then moistened, swells up and gradually diffuses itself in the fluid—the diffusion being more complete if gentle warmth be applied. By means of a powerful microscope the animalculæ, characterised by their long tails, may then be readily discovered.

3. Dried semen dissolves itself in saliva and also in urine, and the animalculæ are found to be not at all altered in their characters.

4. Pure alcohol, and concentrated solutions of potassa, soda and ammonia do not dissolve the spermatic mucus; they cause it to shrink up, and they destroy at the same time the animalculæ. If these reagents be however sufficiently diluted with water, they exert a very remarkable dissolving action on the mucus without destroying the animalculæ.

5. To detect the spermatic animalculæ in spots upon linen, &c. we must take great care that it be not rubbed or squeezed either before or after maceration. By filtering the fluid of maceration, and then examining the deposit that remains on the filter we can generally detect the spermatic animalculæ uninjured, and with their bodies and tails perfect.

6. The presence of the animalculæ may be discovered in the mucus of the

---

* He states that he has been engaged in eleven judicial enquiries connected with the present subject since the beginning of the present year, and that in each of them a microscopical examination, conducted in the manner explained, has furnished *resultats certains*.

vagina, (provided the woman has no morbid discharge) for many hours after sexual intercourse.

7. Dr. *Bayard* says that he has been able to recognise the presence of the animalculæ *a longue queue,* entire and complete in every respect, on spermatic stains of from two to upwards of twelve months old.—*Annales d'Hygiene Publique.*

---

### M. Orfila on the Signs of Death from Hanging.

M. *Orfila* examines the important medico-legal question, how far the state of the genital organs in the male can enable the physiologist to determine whether suspension has taken place during the life or after the death of the deceased. "You know, gentlemen," says M. O. addressing himself to the members of the Academy, "that the presence of zoospermes in the urethra and the congested condition of the genital organs have been regarded by M. *Devergie* as certain signs that the deed, either of suicide or murder, had taken place during life. Now I cannot admit the truth of this idea; for numerous experiments which I have performed demonstrate, in my opinion, its utter fallacy. It is well known that the presence of spermatic animalculæ may be discovered in the urine which has been voided not only immediately, but for several hours, after emission. Now if this be so, we have only to suppose that during the interval death has taken place naturally, and that the body has been suspended afterwards; and then the signs, which M. *Devergie* regards as indicative of his doctrine, will be found present.

But independently of this consideration, I may state that, having most carefully examined the urethra in five persons who had died of different diseases, and were immediately after their decease placed on their backs, I found in all a certain quantity of spermatic fluid in the canal, the animalculæ being even sometimes seen to be still alive. I should add, at the same time, that in several bodies examined more than four and twenty hours after death, the presence of semen could nor be detected: the involuntary discharge of the urine at the close of most diseases may partially account for this absence.

As to the question of the alleged congestion of the genital organs after death from hanging, I may mention that I have repeatedly suspended dead bodies, when these organs had previously been observed to be quite flaccid, and that I have constantly found them after a certain lapse of time to become congested, and in one case even obviously erected.

In a phthisical patient, 30 years of age, the penis increased seven lines in its circumference, and a milky fluid, which oozed from the extremity of the urethra, was found on examination to contain dead zoospermes.

In another case the penis enlarged by at least nine lines, and its cutaneous veins became remarkably dilated.

In a third case, the congestion was so great that the penis was stiffly erected and its veins were quite distended; when the corpus cavernosum was divided, a complete stream of blood flowed out.

It appears therefore from these facts that the opinion of M. *Devergie* is contradicted by experience.—*Memoires de l'Academie.*

---

### M. Civiale on the Treatment of Gravel.

The following remarks are from a memoir which this distinguished surgeon read at a recent meeting of the Academy of Sciences.

"Much attention has of late years been paid to the history of gravel; but most of the enquiries, having been undertaken with systematic prepossessions, have terminated only in false conclusions. For, in truth, the formation of gravel has been almost invariably regarded as being dependent on the mere laws of chemical affinity, and the consequence of this has been that medical men have deemed it quite sufficient in practice to counteract the play of this affinity.

Then again, as soon as it has been discovered that a calculous concretion has been formed, the chief, nay the sole, effort of the practitioner has been to effect either its discharge or its destruction by means of chemical agents, without paying any regard to the organic modifications which gave it birth, or to the influence which it gradually exerts upon the entire system by the mere circumstances of its presence in some part of the urinary apparatus.

What strikes us as especially remarkable in reading the numerous works on calculous disease is, that the authors have generally mistaken, for the essential or primary disease, *one* merely of the effects or results of several morbid states. Hence they have directed their attention only to the removal of the foreign substance already formed, without considering the organic predisposition which has led to its formation; and thus they have allowed their minds to be seduced into a multitude of merely arbitrary conclusions.

Not long ago it was imagined by many writers that calculous disease was scarcely, if at all, known in warm climates, and also that it was of much more frequent occurrence in old men who had been free livers than in others. Now both of these two conclusions have been proved to be quite erroneous by most authentic and trustworthy reports.

It is not surprising that most of the theories, which have been proposed on this subject, should be unable to stand; seeing that they are faulty at their very basis; for the circumstances, which have been viewed as culminant, have in truth little or no real importance in themselves.

The chemical theory of calculous disease can be applicable only at that moment when the urinary secretion has been brought, by a series of morbid actions, to the conditions which determine the formation of gravel. But the question may be asked, what has prepared or paved the way for these conditions? What circumstance or circumstances cause a predominance in one case of uric acid or of urate of ammonia, in another of cystic oxyde, in a third of oxalate of lime, and in a fourth of some phosphate or another?

Now these are questions which it is quite necessary to solve, before we can hope to dissipate that empiricism which hangs round the treatment of gravel. I have attempted the solution; and the manner, in which I have explained the formation of urinary concretions, suggests the elements or principles of that therapeutic practice, which a long and extensive experience has now proved to be so beneficial.

One very fruitful source of the errors of medical men in reference to the success of any particular mode of treatment arises from the circumstance of their losing sight of their patients, and of their mistakingly supposing that they have been permanently cured—whereas, too often, they have left one physician only to go and consult another. Of the truth of this I almost daily see examples.

It is only of recent years that the attention of surgeons has been directed in an especial manner to facilitate the escape of calculi by the urethra. It is a fact of continual observation that, after the operation of lithotrity, fragments of calculi, sometimes of considerable size, will spontaneously be discharged with so little suffering, that the patient is scarcely aware of the occurrence. Now this circumstance, which at first appears to be very strange, is altogether attributable to the diminution of the sensibility of the urethra, in consequence of the preparatory treatment, and to the increase, at the same time, of the contractility of the bladder induced by the manœuvres of the lithotritic operation. The observation of this fact naturally led to the practice of employing the same means to prepare the passages for permitting a transit of calculi which have descended from the kidneys. The truth of the precept has been established, and the practical results from adopting it have been most highly satisfactory.

Hitherto the morbid conditions of the organs, with which the calculi are in contact, or along which they must pass in order to be discharged outwardly, have been almost entirely overlooked. These morbid conditions are however as common as they are numerous; and each and every one of them may present some obstacle to the spontaneous expulsion of the calculus. The chief of these are the spasmodic narrowings and the organic contractions of the urethra, lesions of the prostate gland or of the neck of the bladder, and atony and paralysis of the bladder itself.

The only effect of these diseases, where they are not complicated with calculus, and provided also they are not developed in their extreme degrees, is to cause the excretion of the urine to be tedious and perhaps painful; but in calculous patients their effects are much more serious, for they directly oppose the escape of the calculi and gravelly matter, or, at least, they render its escape difficult and distressing. I have seen a great number of calculous patients, who had tried without any benefit all the therapeutic means that could be imagined. On examining the state of the urethra in many of these patients, I at once detected a state of spasm or neuralgia, under the influence of which the canal had lost most of its pliancy and power of expansion; and by removing this morbid condition, I quickly succeeded in enabling nature to get rid of the gravel within the bladder, although all medical treatment was suspended at the time.

In the case of other patients placed in less unfavourable circumstances, the expulsion of the gravel continues to take place in spite of the nervous condition of the urethra; but this is accompanied with painful sensations, and in some instances it is only after most severe and protracted suffering that the foreign body or bodies are discharged.

The employment of such means as are calculated to relieve the spasm or neuralgia of the urethra will be found very generally to facilitate the expulsion of the calculi. Such, at least, has been the result of my experience in a multitude of cases, where no benefit had been derived from a rigid vegetable diet, enormous quantities of the alkaline bicarbonates, and a long-continued use of the most approved mineral waters.

Contraction of the urethra is certainly not of rare occurrence. The mere presence of calculi in the bladder has a tendency to induce this morbid state; and thus an obstacle is at once presented to their easy exit. When the contraction is more decided, its existence is denoted by special symptoms—such as the frequent desire to pass urine, the obstructions in doing so, the diminution in the size of the stream, &c. &c. The urethra may, however, be very considerably contracted, without there being almost any difficulty in voiding the urine, and yet no calculi, however small, can pass with ease.

It is therefore an important object in the treatment of calculous complaints to counteract all tendency to contractions of the urethra, by the use of bougies. By effecting this, the bladder may possibly be prevented from becoming thickened and contracted, as we usually find it to be in the cases now under consideration.

The prostate gland too is subject to various organic lesions, the common effect of which is to deform the urethra, change its directions, and at the same time deprive it of that pliancy which is necessary to the exercise of its functions. But it is chiefly on the escape of calculous matters that the diseases of the prostate have the most injurious influence. Hence those matters are usually retained in the bladder, and thus gradually become larger and larger, according to the duration of their sojourn, and the tendency of the urine to precipitate its solidifiable principles.

Now, if the attention of the surgeon be not directed to remove this morbid condition of the neck of the bladder, or to counteract its influence, no medical treatment against the gravel can be of almost any avail.

Independently of the direct obstruction, in some degree merely *mechanical*, caused by the enlargement of the prostate, or by any other swelling about the neck of the bladder, there is another, which I would denominate *vital*, and which arises from an increase in the sensibility and contractility of the urethra or of the cervix vesicæ.

This affection is usually connected with an incipient organic lesion of the prostate gland and also with the presence of gravel;—so that there are here two obstacles, dependent one upon the other, and of which one may likewise be produced and aggravated by the very gravelly matter, whose escape it prevents. The plan of treatment, which I have recommended against urethral neuralgias, is usually sufficient to regularize the excretion of the urine; and when once this end is gained, the gravelly matter will in general be expelled without the assistance of art. When indeed the organic lesion is serious, the case is often quite intractable; the spasmodic contractions of the neck of the bladder cannot perhaps be overcome by any means, and then the sand and gravel are retained. Under such circumstances, I have repeatedly had recourse to lithotrity for the purpose of effecting the artificial extraction of the foreign matter, and in this manner I have succeeded in curing a great number of patients, who had been for a length of time fruitlessly subjected to the medical treatment in ordinary use.

Such are the cases in which I have employed with very decided advantage injections of water in a full stream, by means of large catheters provided with ample eyes at the points. Although the gravel is removed from the bladder, it is still very necessary to watch the patient; as we must not forget that there remains at its neck a cause of irritation, proceeding from the morbid state of the prostate gland, and the strangury which this induces:—otherwise the gravel will speedily be reproduced.

Besides the causes hitherto mentioned, which tend to prevent the easy expulsion of calculous matter, there is one dependent on the state of contractility of the bladder, which we now proceed briefly to describe.

It is well known that this organ must have, in order to execute with regularity its functions, a certain degree of energy or tone, and this power be equally diffused over the various parts of the apparatus which act together in expelling the urine. Now it often happens that the bladder is deprived of a portion of this faculty, and that, while there is weakness at one part, there is an excess of contractility at another:—there is spasm of the neck, and atony of the body of the viscus.

Hence arises a double cause of obstruction to the free passage of the urine, and of the almost total impossibility of expelling any calculous matter.

If the disease be not very serious, we may often succeed in relieving such a state of things. For the purpose of diminishing the irritability of the vesical cervix, the repeated introduction of bougies will be found to be the most simple and effectual means; and the atony of the body of the bladder is best counteracted by injections of cold water. In a great number of instances I have enabled patients, by the use of these means alone, to expel all the calculous matter in their bladders with great ease and with little or no suffering.

The three sorts or causes of impediment to the spontaneous discharge of gravel—the narrowings and contractions of the urethra, lesions of the prostate gland, and atony of the bladder—may co-exist in one patient. Although such a complication necessarily adds much to the difficulty of treating the case, we shall often succeed in obtaining the expulsion of the gravel, and thus saving our papatient from calculus, by combining in practice the various modes of treatment, adapted to each, at one and the same time.

It is not only in reference to the expulsion of calculous matter that it is of high importance to remove the different morbid states now alluded to: they likewise exercise an influence, not less powerful but hitherto not appreciated, on the function of the kidneys, and consequently on the secretion of the urine; one effect of which is to modify and alter its chemical composition so much, that the tendency to precipitation is greatly increased. The therapeutic means therefore, which I have pointed out above, not only serve to diminish the inconveniences and dangers arising from gravel, but they are also the most effectual preservatives—in many cases they alone suffice, without the assistance of any medical treatment—against a return of a disease, which may have existed during many successive years.

After what has been stated, it will be readily understood how necessary it is to explore the urethra and bladder in calculous patients; and yet almost all writers have utterly neglected to point out the utility of such a practice, and have given no other rule to guide the young surgeon than that of merely relieving symptoms as they arise. This is one of the causes, which have most contributed to render of no avail the various methods of treatment, which at different times have been recommended.

Every one can easily understand that, if a calculus be too large to traverse the urethra, all medical treatment which tends to promote its expulsion must be useless, if not positively injurious. Besides, the longer that the gravelly matter is retained in the bladder, the more bulky it will become, until at length one or more large calculi are formed—the removal of which can be effected only by lithotomy or by lithotrity. It is therefore of the very highest importance that the disease be detected in its very earliest stage, and that means be used to discharge any foreign matter from the bladder, while it is in small quantities and of little bulk.

Patients themselves have not been without some influence on the stationary condition, in which the history of gravel disorders has for such a length of time languished. The greater number of persons, suffering from these, give themselves up to alarms, of which not a few have become victims. They represent to themselves so frightful a picture of the examinations necessary to ascertain their disease, and of the operations requisite to cure it, that they recoil from the employment of these means, and will submit only to some palliative mode of treatment, during the continuance of which the gravel passes to the state of real calculus.

Among the circumstances, which have most contributed to mislead gravel patients into this error, is the dangerous curiosity of trying to make themselves acquainted with the nature of their disease, by reading all the publications which issue from the press on their disease.

That some medical writers have fostered this pernicious feeling, by addressing their works to the popular reader, is alas! but too true.

On the other hand, the periodical press is found to teem with successive remedies or modes of treatment, which are held out as of infallible efficacy. Every thing is greedily read by the poor patient; and thus not only is he tortured between vain hopes on the one hand, and the terrors of irremediable suffering on the other, but he at the same time is losing much of that precious time, during which his disease, if not relieved, is most assuredly gaining ground.

Such is one of the many evils which spring from the press—too often lauded as the parent of unmitigated good, but which alas! like all other human inventions, is faulty, imperfect, and most pregnant with evil. *La Lançette Française.*

---

### Epilepsy after an External Injury cured by Trephining.

In a recent number of the Italian Journal, *Il Filiatre Sebezio*, we read the following case reported by Dr. *Renzi.*

"A youth, 18 years of age, had been subject since he was ten years old to attacks of epilepsy: they had resisted every mode of treatment, and within the last half-year had become so frequent as to return almost every week. By falling from a scaffold he sustained a fracture of the frontal bone, and also of the left thigh. For five months he was confined to bed. The wound of the forehead suppurated and the injured bone exfoliated. During the whole period of treatment, there was no return of any epileptic paroxysm; and moreover, the patient had quite lost that stupid air so frequent in epileptic patients, and his intellectual powers seemed to have become altogether invigorated. But scarcely was the wound of the head healed than the epileptic fits returned with fresh violence. A seton however being immediately put in the neck, the disease was checked and finally arrested."

The reading of this case reminded M. *Busse* of a case, which had come under his notice many years ago. A youth received some severe blows on the head from his master, whom he had displeased. After the immediate effects of the injury had passed away, the boy became affected with paroxysms, first of chorea and subsequently of decided epilepsy. The frequency of these paroxysms increased so much, that at length the patient had several of them every day. On examining the head, it was found that there was one spot on the vertex which was excessively tender when pressed upon.

An incision was made through the scalp down to the bone; but no unusual or abnormal appearance was perceived. Notwithstanding this, *Hufeland* recommended that the bone should be trephined. During the very process of perforation, the patient was seized with a fit. An effusion—it is not stated *where*—and a fissure of the internal osseous lamella were discovered. The patient not only recovered perfectly from the effects of the operation, but never had any return of the epileptic disease.

His health continued quite good for many years; he married and became the father of a healthy offspring. During a period of upwards of twenty years he had only three attacks of an epileptic paroxysm: each of these attacks had been preceded by indisposition, and had been brought on by mental emotions.—*Hufeland's Journal der Practischen Heilkunde.*

---

### On Diuresis as a Revulsive Action in Diseases of Infants.

Dr. *Simon* prefaces his remarks by alluding to the frequent inactivity, and sometimes the almost complete suspension, of the functions of the bowels and kidneys, while the system of the child is suffering severely from dentition. Whenever the intestinal or urinary excretion is much diminished, the febrile irritation of the system, it is well known, is invariably greater than usual; and, if this state of things be permitted to continue without relief, there is much risk of alarming cerebral symptoms quickly making their appearance. The practitioner will therefore do well to pay particular and uniform attention to the condition of the bowels and kidneys in all diseases of infancy and childhood. The simple question as to the quantity and colour of the urine—and by the bye we can much better trust the report of nurses about the state of the urine than we can about that of the alvine evacuations—will often enable us at once to form a correct opinion as to the general or constitutional health of our patient. As long as the kidneys act freely, there is little or no risk in the symptoms of mere dentition, however severe and distressing these may be. The same remark is, we believe, strictly applicable to the prognosis of most cerebral affections in children. When the urinary secretion is scanty and deep-coloured, the circulation seems to be both oppressed and excited; and the rapid, on some occasions almost instantaneous, mitigation of the alarming symptoms after a copious discharge of water is well known to all experienced practitioners. To promote this critical diuresis, a purgative composed of senna and salts, and then frequently repeated doses of nitre,* are the simplest and most efficient means that can be resorted to.

The chief danger of dentition is referrible to the vascular excitement of the brain. Nor is this wonderful; when we consider that for several successive months there is a continued, and often very severe, irritation in its immediate neighbourhood. The pain attendant upon the cutting of merely one tooth, in our adult years, may teach us to form some idea of the suffering of an infant during the period of its first dentition.

Now it is a common observation that almost all head-aches are most promptly relieved by whatever stimulates the kidneys to throw off a quantity of urine. When this takes place, the system feels at once relieved of a load or oppression which seemed to clog all its energies, and the mind as well as the body becomes more light and vivacious. We are thus led by the experience of our own feelings to anticipate the benefits, which must attend the stimulation of the kidneys in the various affections of children arising from teething.

On the whole, we do not think that there is a more important sign to be attended to in the management of children, during the first two years of their life, than that afforded by the state of the urinary secretion. If nurses and mothers were better aware of this simple, but most valuable, suggestion, the life of many an infant might be saved; for disease would often be detected in its earliest stage, and then might certainly be arrested by the administration of appropriate means. With regard to medical men, we strongly counsel them to make it an invariable rule in their practice to enquire into the state of the urine. As we have already hinted, we can more generally depend upon the reports of mothers and nurses as to the appearances and condition of the urine than of the alvine evacuations, in those cases where we cannot examine the excretions for ourselves.

Nothing will more contribute to relieve the system of the feverish irritation, under which the system of a child suffers during dentition, than a copious diuresis. We should bear in mind too, that independently of the excitement arising from this cause, there is naturally and necessarily a tendency to over action of the blood-vessels in the head during the first year or two of life. The brain, it is well known, is larger then in proportion to other parts of the body than in after years; all the senses are, probably, more acute, the mind as well as the body is rapidly growing, and perhaps scarcely a day passes over without there being made some addition or another to the store of infantine perceptions and ideas. We cannot wonder then that the simultaneous development of so many

---

* Some practitioners are in the habit of adding minute doses of digitalis to the diuretic mixture; and seemingly with good effects. The following formula will be found to answer well.

℞. Potassæ nitratis.......... ℈j.

Aquæ m. viridis.......... ℥ijss.

Syrupi croci.............. ʒij.

Vini antimonialis ........ ʒiss.

Tinct. digitalis............ ♏xvj. M.

A table spoonful to be given every two or three hours.

organs and new faculties should have a powerful influence on the physiological and pathological phenomena which characterize this period of life. To refer all to the excitement arising from the evolution of the teeth, is to take a very partial, and therefore an erroneous, view of this important question. A valuable therapeutic principle is suggested by these considerations; and it is this: that in the treatment of many diseases of infancy we have rather to *regularise* than directly to check or overcome; and therefore that we should most attentively examine the condition of all the functions of the body, in order that we may discover the direction or sense, so to speak, in which nature's efforts are working, and be enabled to assist her in these efforts.

Before closing these remarks, we may very briefly allude to the notable effects which diuretic medicines sometimes exert on the progress of hooping-cough. The administration of nitred drinks and of minute doses of digitalis* seems often to promote the crisis of the disorder in its earlier stages; and, in its more advanced and chronic form, the use of tincture of cantharides has been recommended by Dr. Watts and others, as one of the most efficient antidotes.† The excitement of the urinary viscera produces a powerful revulsion on the neurosthenic condition of the gastro-pulmonary apparatus, and thus seems to act as a derivative of the morbid action.

In fine, the kidneys become, in numerous cases of disease, the seat of an active eliminatory process, of which the skilful physician will avail himself in the treatment of dentition and of various other affections to which children are especially liable during the first two years of life.—*Bulletin General de Therapeutique.*

---

### ON THE INTERNAL USE OF ACETATE OF LEAD IN ANEURISM OF THE AORTA, &c.

Aneurism of the aorta is very properly considered as an incurable disease; for in all the cases where a cure has taken place, this has been the result of the efforts of nature, and certainly not the direct effect of the remedies employed. We do not mean by this assertion to disparage or condemn the use of all therapeutic means in the treatment of this formidable affection; far from it; judicious medical regimen, as regards not only the administration of drugs and the regulation of the diet, but also the general management or discipline of the entire system, mental as well as corporeal, may powerfully contribute to accomplish the desired end, by encouraging the formation of coagula within the sac, and removing all the impediments to this curative process. The mode of treatment so strongly recommended by *Valsalva,* that usually it passes by his name, and which consists, it is well-known, in frequently repeated abstractions of blood and in continued rest and quietude, appears to us to be of very questionable propriety. For the effect of it must surely be to attenuate the condition of the blood itself, and therefore at once to diminish its coagulability or tendency to deposit its

---

* Dr. Simon recommends the external use of tincture of digitalis rubbed, as a liniment, on the abdomen.

† A favourite formula of some physicians in certain chronic cases of hooping-cough is the following:—

℞. Tinct. cinchonæ.......... ʒiij.

Tinct. lyttæ ............ ʒj.

Tinct. camphoræ comp..... ʒij.

Mist. camphor........... ℥ijss. M.

Capiat coch. j. magnum ter in dies.

fibrinous constituent—the very property by the operation of which the curative process is to be effected. M. *Dupuytren* had for many years abandoned the practice of frequent bleedings in the treatment of diseases of the heart and large blood-vessels. Blood was drawn only for the purpose of relieving present distress and existing congestion. It seems therefore to be more rational to make use of any means—if such really do exist—which have the effect of retarding the force of the circulation, without at the same time altering the qualities of the blood. The late Baron *Dupuytren* and some other medical men were in the habit of occasionally using the acetate of lead, as an internal remedy, with this view. MM. *Dusol* and *Legroux* have collected together the data of several cases where this saturnine preparation seemed to produce good effects in cases of aneurism of the aorta.

It is to be regretted that the reports are not sufficiently minute to satisfy the medical reader in every respect; but it cannot be denied that they are sufficient to justify the conclusion that the remedy deserves to be more frequently used than hitherto it has been.

The following is the statement of one of the cases:

A man, 37 years of age, was admitted in May, 1829, into the Hôtel Dieu, and under the care of M. *Dupuytren,* with a pulsatile tumor extending from the right side of the sternum in the direction upwards. Three years before the date now mentioned, he had suddenly experienced, while lifting a heavy weight of timber, a sharp pain on the right side of the chest, accompanied with difficulty of breathing. He continued however regularly at his work for the next fifteen months; but the oppression gradually increasing, and being now attended with severe headaches and acute pains in the right shoulder and extending upwards along the neck in the direction of the great blood-vessels, he had been bled once or twice from the arm; but this treatment had afforded partial and temporary relief.

At the end of several months, a tumor was perceptible outwardly: and this gradually attained the size of a large egg. As this outward swelling made its appearance, the dyspnœa became less troublesome, until at length it almost entirely ceased for a time. The distress of the patient however, soon returning, he applied for admission into the hospital. The tumor was found to pulsate isochronously with the beats of the heart; the skin was somewhat reddened and seemed to have become attenuated; there was frequent cough and dyspnœa—which was sometimes so distressing as to prevent the patient from laying down—congestion of the features, occasional difficulty in deglutition, and tendency to most annoying dreams during sleep. After a small bleeding, the patient was ordered by *Dupuytren* to take a pill, containing one grain of acetate of lead, twice in the course of the day; the number was gradually raised to six in twenty-four hours. Within three weeks, the size of the swelling was very sensibly diminished; the same treatment was therefore ordered to be continued, and the part was to be kept covered with compresses, wetted with a saturnine wash. The dose of the pills was raised to eight and then ten, each containing one grain of the acetate, a day during the month of June; and as the patient at this time began to suffer vomiting, the medicine was omitted for about a week. It was, however, then resumed and persevered in for another fortnight. The patient at this time left the hospital in all respects wonderfully relieved: he was advised to continue the same treatment for some weeks longer.

The second and third cases reported in the memoir of MM. *Dusol* and *Legroux* are so very similar to the one now briefly narrated, that it is unnecessary to particularise their details. In all the three cases, not only did the outward swelling very notably subside, but also the respiration experienced such decided relief, and this too long before there could have been any decided diminution in the pressure of the tumor on the lungs or trachea, that we deem it probable that the remedy acted directly upon the organs of circulation.

The influence of acetate of lead in hæmoptysis, and in some other forms of internal hæmorrhage, is very generally admitted. How does it act in such cases ?—it seems probable that it is by a directly sedative operation on the heart, and thus removing, or at least mitigating, the *engouement* and congestion of the pulmonary tissue. The acetate of lead has been used with advantage to check the amount of purulent expectoration in phthisis, to arrest all excessive evacuations from the bowels, to moderate the superabundant flow of milk, &c. : it seems in short to be a very general astringent.

The study of the rationale of remedies—or in other words the *science* of therapeutics—is a subject of first-rate importance to the physician. Indeed it affords by far the best means of improving the practical part of our profession ; and this, after all, is the end and scope of our labours.—*Archives Generales.*

---

### ON HYDROCELE OF THE NECK.

Those encysted tumors of the neck which have been sometimes designated hydrocele of the neck, although mentioned in the writings of some of the older authors, had attracted little notice, until M. *Maunoir* of Geneva published, in 1825, his work entitled " Memoires sur les Amputations, l'Hydrocele du Cou, et l'Organisation de l'Iris." M. *Delpech* subsequently narrated, in his Clinique Chirurgicale, two cases which he had successfully treated by operation. Messrs. *Lawrence, O'Bierne, Heidenreich, Beck,* &c. have since published the reports of a few additional instances of this rather rare disease, and have thus contributed to enlarge our knowledge of its true characters.

According to the researches of MM. *Fleury* and *Marchessaux,* in the August Number of the Archives Generales de Medecine, there are two sorts of these tumors, which may be distinguished from each other by the difference in their anatomical characters.

The *first* are those which are developed in the actual tissue or substance of the thyroid gland. They are sometimes superficial, at other times deep-seated : they correspond to the cellular or thyroidean serous goitre of *Beck* and *Heidenreich,* to the hydrocele of the neck of *Maunoir,* the hydro-bronchocele of *Percy,* and the encysted goitre of other writers.

The *second* are developed in the common cellular tissue of the neck, at a greater or less distance from the thyroid gland, and, according to some authors, in the cellular texture of this gland itself. These have been denominated by *O'Beirne* hydrocele of the neck, cystic tumors by *Boyer* and *Dupuytren,* fibro-serous cysts by *Delpech,* and hygroma cellularis by several German surgeons.

MM. *Fleury* and *Marchessaux* have given a very minute description of the anatomical peculiarities of these two sorts of tumors. Those, which more properly appertain to the thyroid gland, had already been ably treated of by MM. *Andral* and *Beck.*

The other kind, or such as are developed in the cellular tissue, are genuine encysted swellings ; the cysts of which are formed by the progressive development of a fibrous tissue, which, as Bichat first demonstrated, exhibits many of the characters of serous membranes. The skin over them does not usually undergo any change, except perhaps when it adheres very firmly to the subjacent cyst ; and then it becomes so very thin, from the absorption of all the fatty matter, that the minute bloodvessels can often be seen through it. At other times, the cyst is connected with the surrounding parts only by very loose cellular-tissue, so that it remains very moveable. The parietes of the cyst are usually firm, little or not at all elastic, and thickened. The thickness is sometimes remarkable : in one case the anterior wall of a cyst was found to be nearly an inch in thickness. Not unfrequently within their cavity are found laminæ of a cartilaginous and even of an osseous formation.

These characters are always the more decided in proportion to the length of time that the swelling has existed. The internal surface of the cyst, when its aspect is not completely modified by the transformation which it has undergone, exhibits a white, reticulated, appearance, not unlike to the inner surface of the ventricles of the heart, or of the urinary bladder in certain cases. This surface is coated throughout with a pseudo-serous membrane, which invests all the ridges and furrows, and which some anatomists have described under the name of internal lamina of the cyst. Its thickness, colour, and consistence vary much in different cases: in some it is smooth and of white colour, while in other cases it is more or less red, and resembles a softened mucous membrane. Occasionally pieces of this lamina become detached, and, floating about in the fluid of the sac, have been mistaken for hydatids.

The encysted tumors of the neck have been met with in both sexes, and at almost every period of life: sometimes they are congenital. Their growth is usually very slow; and in most cases no cause can be assigned for their appearance. Occasionally they have been observed to enlarge rapidly after a catarrh, and also after an accouchement.

If allowed to attain a large size, they impede the respiration, and even the deglutition, as well as the circulation in the neck and head.

The fluctuation of their contents is generally more easily perceptible in the earlier stage of the disease—provided the tumor be sufficiently prominent—than when it is more advanced, in consequence of the parietes of the cyst gradually becoming thicker and more resisting. Hence mistakes in diagnosis have occasionally been made by the most experienced surgeons, and cases of hydrobronchocele have been considered as examples of goitre, or of some other solid growth. We may also mention that the tumor may sometimes be mistaken for an aneurism of the carotid artery, in consequence of its communicating the pulsations of this vessel; but a careful examination of the case will generally enable the surgeon to discover that the swelling experiences a lifting up or rising *en masse*, and not those movements of alternate expansion and retrocession, which are characteristic of a genuine aneurism.

With respect to the different methods of treating hydrocele of the neck, authors very generally agree in regarding *puncture* as a mere palliative means, and as one moreover not always free from inconveniences; and they condemn *injection* of the sac, as uncertain in some cases and highly dangerous in others. The use of a *seton* or something analogous, as a canula, tent, &c. appears to be the practice most extensively approved of.

In one of the cases, which occurred to *Dupuytren*, the sac refilled to its former size within a short time, although a seton had been in it all along; a canula of elastic gum was therefore introduced and left in the lower opening, so as to permit a ready escape to the contents of the sac, and at the same time to allow the occasional injection of emollient and detersive washes.

MM. *Fleury* and *Marchessaux* also record a case, in which the same method was adopted with success, after various other means had been ineffectually tried.

In a case, which occurred in a girl 17 years of age, M. *Jobert* punctured the tumor three times successively, the fluid having re-accumulated quickly after each operation. After the third puncturing, some alcoholised water was injected into the sac, and then a seton was introduced for the purpose of keeping the wound open. At the end of a week, suppuration was but imperfectly established, and there was only a slight serous oozing, when the seton was removed. An elastic-gum canula was therefore substituted for the seton: this was kept in the opening, and gently stimulating injections were repeatedly passed through it. After the lapse of two months, the swelling, which had been of an immense size, was not bigger than an egg; and four months later, all that remained was a small kernel or lump, which was entirely indolent.

M. *Flaubert*, of Rouen, has published a case which he successfully treated by *incision*—a method which has succeeded in the hands also of MM. *Delpech, Morelot, Lemaire*, and which an Italian surgeon has in one case combined with the use of the seton.—(*Annali Universali*. Feb. 1838.)

*Excision* has been repeatedly adopted with complete success. In three cases *Beck*, after having laid open the tumor by an incision, excised a portion of the cyst, which was not connected with the thyroid gland. This method hastens the process of suppuration, and does not seem to be attended with any inconveniences, when precaution is taken to avoid wounding the substance of the thyroid gland.

The *extirpation* of the entire sac may be adopted with advantage, when the cyst is small, superficially seated, and not firmly adherent to the thyroid gland, or to any other of the neighbouring important parts.

M. *Jobert* practised it with success in the case of a woman, 30 years of age, in whom the tumor was of the size of a hen's egg, and was smooth, hard, and so resisting, that it communicated no feeling of fluctuation; the wound healed up by the first intention, the twisted suture having been employed to retain its lips together. But we must refer our readers, who may wish to make themselves acquainted with the particulars of the various cases on record, to the original paper of MM. *Fleury* and *Marchessaux*, and will now extract only the following conclusions, which they have deduced from their enquiries.

1. The encysted tumors of the neck may be arranged, according to their anatomical position and relations, in two classes—one comprising such as are developed in the substance of the thyroid gland, and the other, all those which are developed in the cellular tissue of some portion of the neck.

2. The *first* set seems to be attributable to the hypertrophy of one or more of the cells of the thyroid gland, and, if so, cannot be regarded as encysted tumors, in the strict sense of the phrase; whereas in the *second* set, the sac of the tumor is a genuine cyst of a sero-mucous nature.—*(Delpech.)*

3. This distinction is important both in a diagnostic and in a therapeutic point of view.

4. The tumors of the first class may be readily mistaken for genuine goitre; and those of the second for chronic abscesses, lymphatic swellings, or aneurismal swellings.

5. Encysted tumors of the neck, whatever be their nature, should never be left to Nature; they always require surgical assistance for their dispersion.

6. Among the various methods of treatment, which have at different times been proposed, that of nearly puncturing, and that of injecting the sac, appear to be the least trustworthy and advisable. The employment of a seton, after a free incision has been made, has been found useful in tumors of the first class, by inducing and keeping up a long and abundant suppuration, and thereby causing a melting down of the hypertrophied and swollen tissues; also in all cases where the cyst was multilocular, by giving a free discharge to the contents, and preventing an accumulation in any of the cells. On the whole, it may be laid down as a therapeutic principle, that the incision of the swelling and the subsequent employment of some means to establish suppuration in the cyst, seem to be the best method of treating almost every encysted tumor of the neck, whatever be its origin or seat.

The complete extirpation of the sac may be recommended when this is not very large nor closely adherent to the surrounding parts.—*Archives Generales de Médécine.*

---

### Hints on the Treatment of Wounds after Operations.

The following brief passages are extracted from a letter which M. *Phillips* of Liege has recently addressed to M. *Baudens*, the distinguished chief surgeon of the French army in Africa.

. . . . After an amputation in a joint, the flaps should be brought and kept together by a few stitches, in order to promote union by the first intention; but as adhesion rarely, or perhaps never, takes place between the surface of a cartilage and the contiguous flesh, we should not only remove as much of it (the cartilage) before closing the wound, but also place a small portion of lint upon it, so as to encourage suppuration at this point, while the rest is retained in exact contact. The annals of surgery afford scarcely one instance of complete and perfect union, without suppuration, of a wound after a disarticulating amputation.

The disarticulation of the femur performed by the late M. *Delpech*, where the wound healed almost immediately, is perhaps the most fortunate case on record. The patient had suffered from disease of the femur for nearly 19 years, and he was falling into a state of marasmus. The crural artery being first tied, the chief flap was formed from the muscles on the inside of the limb; the head of the bone was then easily disarticulated, and the operation quickly finished. The two flaps were kept together by numerous stitches, which traversed the integuments only.

On the sixth day, there was a serous oozing from the wound, and on the ninth, a very slight suppuration. On the 28th, the cicatrization was complete; but about the end of the second month, a white substance presented itself; this was the cartilage of the joint. It was immediately removed, and the wound speedily healed.

Such an occurrence—as that of the cartilage making its way to the surface with no local or general disturbance—is excessively rare, and should not influence our practice much. We are therefore of opinion, on the whole, that whenever an amputation is performed at a joint, an issue or opening should always be left opposite to the cartilage, while the rest of the wound is retained together by sutures.

Even after ordinary amputation, where a bone has been sawn across, some surgeons have recommended that the same practice be pursued; but, without positively admitting the propriety of so general a rule, it is worthy of remark that, when the two ends of a bone are sawn off in cases of ununited fractures, not only is there a better chance of a union taking place, but this union is always much firmer and more solid, when suppuration has been established at the point of junction, than when immediate adhesion of the wound has occurred. Such was the opinion of M. *Delpech*; a high authority certainly on all subjects of practical surgery.

. . . . Numerous statistical reports of the mortality after amputations of the larger limbs have been published of late years; but, from the imperfection of their details, such as the mode of dressing after the operation, the use or not of sutures, &c. &c., it would be unsafe to draw any positive inferences. The success of M. *Percy*, after the battle of Neubourg, deserves however to be alluded to, in consequence of certain circumstances. There was such a want of many of the most necessary articles, as lint, ointment, and bandages, in the French camp, that all the patients were dressed indiscriminately with merely cold water. It is probably to this very circumstance that the great success of the surgeons, in the results of their operations, was attributable on that occasion. For some years past, the same practice has been adopted by Mr. *Liston* at the London University hospital, and M. *Phillips* has of late universally followed it with very marked benefit, after almost all his operations.

In all severe injuries of, as well as after operations on the eye, M. *P.* always subjects the part to the action of cold water for several days. Also after the extirpation of tumors, when the lips of the wound have been brought and kept together by several stitches, compresses wetted frequently with cold water are laid invariably upon the part. M. *Phillip* mentions that in such cases he generally unties the ligature at the lower angle of the wound on the day after the operation, for the purpose of giving exit to any bloody or other kind of discharge, and after removing this, he again secures it. On the third or fourth day he removes one ligature, and another on each day successively; continuing the use of the cold applications all the time. M. *P.* trusts to *torsion* of the arteries to arrest the hæmorrhage, after many such operations. There is very little doubt that this simple expedient, if really sufficient as a hæmostatic means, must contribute very materially to the speedy union of wounds, as each ligature on a blood-vessel must necessarily give rise to a point of suppuration and to a minute sinus or fistula extending from the tied vessel to the surface of the wound.—*Bulletin Med. Belge.*

---

### On the Advantages of Delay (Expectation) in the Treatment of Comminuted Fractures.

The great aim of the surgeon consists, or ought to consist, in preventing the necessity of any cutting operation, and in curing his patient without having recourse to the knife. Every operation, it has been well remarked, may be considered as an opprobrium of the surgical art, and as an admission on the part of the surgeon of the inefficacy of his own skill. Influenced by the truth of this sentiment, we propose to endeavour to point out the advantages of *expectation* in the management of certain severe injuries of the joints and limbs. By this term we wish to express a prudent and well-considered delay for several days after the receipt of a severe accident, and during which time a skilful management of all the symptoms, constitutional as well as local, is perseveringly pursued—so that on the one hand a fair chance is given to Nature to repair the damage, and on the other hand, that the health of the patient is not unwisely risked too far before any mutilating operation is resorted to.

It is perhaps the more necessary to call the attention of surgeons to this most important subject in the present day, as there seems to have been, for many years past, a tendency to rather precipitate conduct in the treatment of severe and complicated injuries of the limbs, by having recourse to immediate amputation of the extremity. Encouraged by the precepts and example of our military surgeons—who, it is to be remembered, are often under the painful necessity of amputating at once in cases where, in more favorable circumstances, the limb might be saved by delay—and misled by reports and statistical details of many writers who have certainly exaggerated their own success, practitioners seem to have forgotten the advantages of a *sage reserve.* Certain it is, that the more experienced a surgeon becomes, the less hasty is he to lop off limbs at once, and the more inclined he is to give the system a fair chance of repairing the injury—without however delaying an operation too long, if such be absolutely necessary.

The judicious employment of local and general bleeding, of refrigerant drinks, of low diet, &c. in robust and plethoric constitutions, and of opiates and other anodynes, along with these means, in such as are feeble, lymphatic, and nervous, will often serve to prevent, or at least to mitigate, the severe symptoms which follow as a matter of course, every serious injury or wound. At the same time, the free incision (*debridement*) of such parts as may be tense and confined, the extraction of splinters which are always a cause of much pain and irritation, the resection of any protruded bones, the adjustment of opposite surfaces by means of stitches or bandages, the continued affusion or irrigation of cold water on the seat of the injury, the employment of emollient or resolving remedies according to existing indications, the use of the starched bandage (*bandage amidonné*) as soon as the first or inflammatory accidents have passed away, and the administration of internal or external revulsives when any complication supervenes—these means, if judiciously practised, will in very many cases enable the surgeon to conduct his patient through numerous dangers and difficulties, and restore him to health without having undergone any loss or mutilation. The salutary effects of one or two large blood-lettings, in cases of severe injuries of the limbs, are well known to most practical surgeons; many an unpleasant accident might be avoided by the prompt and decided use of the lancet.

In reference to the application of leeches in such cases, it may be useful to state that they should be applied not to the immediate vicinity of the injury, but at some distance from and above its seat, and if possible near to the point where the smaller veins terminate in the larger trunks.

The irritation caused by leech-bites close to a contused or lacerated wound has often caused serious mischief.

We have already alluded to the advantages of removing any tension in the wounded part by means of one or two free incisions. Some surgeons have of late years questioned the propriety of this practice; but fortunately they have made few converts to their opinion, and the value of the precept is now very generally acknowledged. In many cases we should not wait until the inflammatory tension takes place before we have recourse to this practice, as it is much easier to prevent than to relieve the mischief.

The following are said to be the good effects of a continued irrigation of the injured part with a stream of cold water. "It arrests inflammation, checks the swelling of the tissues, soothes pain, lulls sympathetic irritations, and stifles any sanguineous reaction; the tissues bathed, as it were, in a permanent bath, become pale and colourless; their vessels no longer receive more blood than is necessary for the support of their organic life; suppuration is established slowly and scantily, and the reparative process is not disturbed by any violent excitement either in the part itself or in the system. The water should be of the ordinary temperature; and the limb should be placed in some apparatus which protects the bed from wet, and the parts, which are not exposed to the current, should be enveloped in warm flannel. By attending to these precautions, the risk of any pulmonary or abdominal irritation will be prevented, and the irrigation may be safely continued until all chance of inflammatory excitement has passed away."

In some cases, however, as when there is any tendency to pneumonia or enteritis present, or when the injured parts have been so severely bruised as almost to have lost their vitality—the use of cold affusions is inadmissible. Then we should have recourse to opiate cataplasms, which are especially useful when the process of suppuration is commencing.

With respect to bandages, they cannot be too simple and lightly applied at first. Whenever the risk of inflammatory action is over, and the use of the cold irrigation is therefore unnecessary, there is no remedy so useful in promoting the cicatrization of any wound and the solidification of the callus between the fractured ends of the bones, as the employment of the starched bandage or roller.* It has the great advantage of giving such equable support to the

---

* This—the *bandage amidonné*, which has for the last few years been so highly recommended by MM. *Seutin*, *Velpeau*, and other continental surgeons, as constituting by far the best immoveable case for fractured limbs—is merely a common roller soaked in starch mucilage before it is applied. When it becomes injured limb that the patient may move it about in bed, or even walk upon crutches without any danger; and thus much of the sufferings, which are almost inevitable upon long confinement, is prevented. Moreover it is light, easily applied, and when once adherent is not liable to be displaced. It requires renewal only when, from the subsidence of the swelling of the limb, it has become rather loose around it, or when it is much soiled by the discharges. Openings must be cut in the bandage over the seat of any wound, so that this may be regularly dressed. We regard the employment of the continued irrigation of the injured part and of the subsequent application of the *starched bandage* as two of the most valuable acquisitions which the art of surgery has obtained in modern times, and as destined to introduce, in the treatment of comminuted and complicated fractures and such-like injuries, a most beneficial revolution.

As to the sympathetic or constitutional phenomena, which usually attend all very severe accidents, no general rules can be given how they are to be obviated or relieved. The surgeon's duty is to question, as it were, every organ, so that no symptoms, however obscure and latent, can escape his notice. It is unnecessary to insist upon the prompt adoption of appropriate treatment whenever any signs of an internal phlegmasia make their appearance. But, even under such circumstances, depletions and evacuations must not be carried too far; for it should ever be kept in mind that there is a time for the use of cordials and tonics as well as for that of antiphlogistic remedies.

In illustration of the foregoing remarks, the following cases will perhaps be read with interest.

A sailor was struck in the arm by a large splinter of wood, which had been broken off by a cannon-ball. The lower end of the left humerus, close to the condyles, was broken in several pieces, and there were two contused and lacerated wounds about the joint: the capsular ligament also was found to be ruptured. The two wounds were made into one by a transverse incision; the limb was placed in an easy extended position, and kept constantly irrigated with goulard water; the patient was bled twice in the course of the first day; and an opiate mixture was administered occasionally. On the third day, feverish symptoms setting in, another venesection was practised, and the same mode of treatment in other respects was ordered to be continued. On the seventh day suppuration commenced, and opiate cataplasms were therefore substituted for the cold irrigation. The purulent discharge became greater and greater, and hectic symptoms began to make their appearance. But these gradually subsided, when all the sequelæ of bone were discharged, and then the process of solidification and union went on favourably. In spite of all the endeavours of the surgeon to preserve some degree of motion in the joint, it became anchylosed. The arm however, although stiff and motionless at the elbow, was of the greatest service to the patient, when he was discharged cured after six months' treatment.

In the management of wounds of the elbow-joint, M. *Reynaud*, chief surgeon of the hospital at Toulon, insists much on the good effects of frequently changing the position of the limb, and of moving it about in various directions towards the close of the treatment, so as to prevent the occurrence of complete anchylosis.

*Case* 2.—A sailor on board the *Trident* had his fore-arm crushed between a

---

dry, it forms a firm case round the limb, so that the patient may move it about, or walk on crutches without any risk of displacing the bones. An abstract of a paper by M. *Velpeau*, on the use of this and other kinds of agglutinative bandages, in the treatment of fractured limbs, will be found in the Medico-Chirurgical Review, for July 1838.

carronnade and one of the yards of the ship: both the radius and ulna were broken in several places, and the soft parts were severely bruised and lacerated. By means of one or two incisions all tension of the injured parts was relieved, and a free opening was made for any splinters of bone to escape. Several of these were removed at the time, and the limb, being placed in a fracture box, was kept constantly irrigated with cold water, by means of a syphon which communicated with a jug at the side of the patient. He was bled twice in the course of the day, and cooling febrifuge medicines were administered. The progress of this case was most satisfactory. Several pieces of bone were discharged, and the purulent secretion becoming gradually less in quantity, the several wounds cicatrised. It was now found that a false articulation existed between the fractured ends of the radius, which it had not been possible to retain together in strict contact.

By, however, surrounding the limb at this part with numerous strips of plaster and then enveloping it with the starched bandage—which were kept on for forty days—a union took place. When the patient left the hospital, the fore-arm, although far from retaining its normal freedom of motion, was serviceable for almost every exertion.

The *third* case is one of extensive wound in the knee-joint.

A sailor fell into the hold of a vessel among pieces of broken glass, &c. One of the fragments had penetrated into the left knee-joint, and had caused a very large wound immediately below the patella. This bone was retracted upwards nearly three inches, in consequence of the complete division of the patellar ligament. The internal lateral ligament also was partially cut across, and the capsular ligament of the joint was laid open so extensively that the articular surfaces of the femur and tibia were quite exposed. A small fragment of glass was actually imbedded in the substance of the crucial ligaments. This was removed, and the lips of the wound were brought together, the oozing of blood being stopped by the application of cold water. Wishing to save the limb if possible, M. *Cabissol*, the naval surgeon in attendance and the writer of the preceding remarks, adopted the following practice. The limb was laid upon a long splint which extended from the pelvis to the heel. The wound was united by means of four stitches, assisted by a bandage. A continued stream of cold water, by means of a syphon immersed in a jug placed above the patient, on the joint was perseveringly kept up, and the patient was bled from the arm. Next day sixty leeches were applied at some little distance from the joint, and the bleeding was repeated from the arm. From this date, there was no swelling, pain, nor fever, and by the twelfth day the wound was found to be cicatrised.

On the 22nd day, the cold irrigation was discontinued, and the joint was enveloped in the folds of the starched bandage, so as to retain it perfectly motionless, and promote anchylosis, the occurrence of which M. *Cabissol* considered to be inevitable. The patient was then permitted to move about on crutches. At the end of the sixth week, the knee had recovered its normal size, the patella had nearly descended to its right place, the divided ends of the ligament were united by a plastic nodosity, and there was still a slight power of flexion in the joint, so as to give grounds for hope that the anchylosis might not be complete.—*Bulletin General de Therapeutique.*

---

### Lumbrici discharged from an Umbilical Abscess.

A youth of a leuco-phlegmatic temperament, had been long confined to his bed with all the symptoms of *tabes mesenterica*—such as tumefaction of the abdomen, irregular appetite, feeble digestion, and an almost constant fever. He had been in this state for upwards of a twelvemonth, when he began to experience sharp pricking pains over the left portion of the transverse colon, at about four inches from the umbilicus. In the course of a fortnight these pains became more severe; a circumscribed swelling made its appearance about the umbilicus, and this in the course of a short time proved to be an abscess. It broke of itself, and gave issue to a quantity of purulent matter. On the fifth day afterwards, a *lumbricus* worm escaped from the wound; and a few days subsequently another, and then another, so that at length five of these entozoa were discharged. Along with the last one, there was an issue of matter, which had a distinctly stercoral fætor. No unpleasant symptom supervened; the abscess gradually healed up, and eventually the patient was restored to perfect health.—*Il Filiatre Sebezio.*

---

### RUPTURE OF THE TENDONS OF THE TRICEPS AND RECTUS FEMORIS MUSCLES.

A man, 74 years old, fell forwards when mounting a flight of stairs and his left thigh struck against the edge of one of the steps: he could not rise.

Upon examining the limb, it was at once obvious that the common tendon of the triceps and rectus muscles was completely ruptured across immediately above the patella: the distance between the two separated ends was so great as to admit four fingers. The patient had lost all power of moving the limb in any direction. It was placed in an extended position, such as is adopted in treating fractures of the patella. In spite of some troublesome symptoms during the first and second weeks, the progress of the case was eventually very satisfactory; for at the end of two months the re-union seemed to be perfect, and the patient could raise the limb with tolerable ease from the ground; and, in three or four months more, he could walk up and down stairs without any difficulty or appearance of limping. The old gentleman died from an attack of apoplexy in the course of the following year, and Dr. *Martini* his medical attendant, was permitted to examine the body. He found that the re-union of the two ruptured ends of the tendon was effected by an intermediate semi-ligamentous substance, which was not more than an inch in breadth: there were no abnormal adhesions; but two or three very distinct cicatrices on the capsule of the knee-joint proved that it too must have been partially ruptured in more than one spot.—*Medicinische Corresp. Blatt.*

---

### TREATMENT OF POISONING BY ARSENIC WITH STIMULANTS.

*Royal Academy of Medicine, 30th July.*

M. *Ollivier* communicated, in his own name and in the names of MM. *Amussat, Bouillaud, Husson,* and *Lecanu,* a report upon a memoir of M. *Rognetta,* in which the author endeavoured to establish the comparative value of antiphlogistic and of stimulant means in the treatment of poisoning by arsenic. M. *Rognetta* had performed numerous experiments on dogs, the results of which went in his opinion to prove that the latter remedies were to be preferred; and that bleedings, &c. are in truth hurtful, as had been asserted in the writings of *Rasori* and *Giacomini* of Italy.

There were three sets of experiments; in one, a solution of arsenic was injected into the cavity of the peritoneum; in the second series, the salt in a state of powder was introduced into the cellular substance of the neck; and in a third set, a solution was injected into the stomach of the animal, and vomiting was prevented by tying a ligature round the neck.

1. All the dogs, in the first series of experiments, died in from 9 hours to 4 days, according to the strength of the solution that was used. Those, which were bled immediately afterwards and in which the venesection was repeated, died in a shorter time than either the animals to which tonics—consisting chiefly of a mixture of broth, brandy and wine*—had been given, or those to which nothing was done or administered.

2. In the second series of experiments, two dogs, to which nothing was done, and also the two dogs to which stimulants were given, died; whereas two only out of the six dogs, which had been freely bled, died: the period of death in all varying from 8 to 12 hours after the experiment.

3. In those dogs, into whose stomachs the arsenical solution (from 6 to 14 grains) was injected, the mortality was as follows: of thirteen, which were bled once or several times, eleven died; of thirteen, which were treated with stimulants and narcotics, only five died; and of nine, to which nothing was either done or given, eight died.

It must be here noticed that of the eight dogs which recovered under the use of the stimulating and narcotic treatment, we have only insufficient data in reference to some of the most important features of all such cases—as, for example, whether vomiting took place, and if it was frequent and energetic. In four only out of the eight animals did vomiting—at least we are not informed to the contrary—occur. This want of information is certainly to be regretted; as, in all experiments upon poisonous substances, it is of the very first importance to know whether the poison was entirely retained, or whether it was partially rejected by vomiting—more especially when the poison has been administered in a state of solution. Arsenic, when it has been taken for the purpose of self-destruction, is usually taken in a solid form, mixed up with some thick pudding substance. It thus attaches itself to the parietes of the bowels, and is much less easily detached and rejected than if it had been in a state of liquid.

Forty-seven dogs in all were subjected to the experiments; of these 19 were bled, 20 were treated with stimulants, and 9 were left without anything being done. Of the first, 19, sixteen died; of the second, 20, eleven died; and of the last set, 9, eight died.

In addition to the lesser mortality among the dogs which were treated with stimulants, it was observed that they more quickly recovered from the effects of the poison than those which had been bled.†...... The reporter, M. *Ollivier*, in conclusion proposed that M. *Rognetta* be thanked for his interesting communication, and be requested to continue his researches.

M. *Orfila* spoke to the following effect.

Before entering on the present discussion, I can assure you, gentlemen, that although an author on toxicology I am quite disinterested and free from all prejudices one way or the other. I have not advocated the adoption of blood-letting and the use of other antiphlogistic measures either indiscriminately or under all circumstances of poisoning with arsenic; for it will be found on consulting my work, that, after quoting the cases related by some writers in favour of this mode of treatment, I have simply said, "I regard it as useful whenever symptoms of inflammation have made their appearance."

In estimating the value of any curative means in cases of poisoning, whether from arsenic or from other acrid substances, it is most necessary to remember that there are two periods in the treatment: in the *one*, we endeavour to expel

---

* If this was rejected by vomiting, more was again given; and if the vomiting was renewed, it was then given as a lavement. Occasionally some laudanum was added to the mixture.

† It results from the experiments of M. *Magendie* and other physiologists, that the detraction of blood encourages absorption.

or withdraw the poison from the stomach, and to neutralize by chemical antidotes* what may remain behind; in the *other*, our aim should be to counteract and subdue the symptoms which arise, whether of inflammation or of debility and exhaustion, of excitement or of stupor. These symptoms too, it is well known, vary much in different circumstances. In one case vomiting takes place very early, while in another it does not come on for some time; in one case the stomach is full at the time when the poison was swallowed, while in another it is empty; and so forth.

Now it is scarcely possible to imitate all these varying circumstances, when we give a poison to dogs in the way of experiment. If you give a feeble dose, they will generally vomit it up at once—and vomiting occurs in dogs with great readiness—and be quickly well again, so that scarcely any symptoms of poisoning are observed. This indeed is so frequently seen, that M. *Virey* went so far as to put the question whether arsenic was really a poison to dogs.

But to proceed to the statement of particular facts, I may mention that during the last five years eighteen cases of poisoning by arsenic, where bleeding was resorted to with success, have come under my notice; and M. *Villard* has given me the particulars of other five cases which were equally benefited by the use of depletory measures.

I have also collected the reports of nineteen cases, which have been published in the English journals; carefully noting, among other circumstances, the occurrence or not of vomiting, the frequency with which bleeding was performed in each, &c. &c. and I find that one only of the nineteen patients died. It may also be noticed that the symptoms induced by an over-dose, or by a too-long continued use, of an arsenical preparation in the treatment of cutaneous and other diseases, are generally of an inflammatory or pyretic character, and require the employment of antiphlogistic remedies. All these facts clearly prove that bleeding and other similar measures are highly useful in certain cases, at least, of arsenical poisoning.

Now to come to M. *Rognetta's* experiments, we observe that all the dogs, in which a solution of the poison was injected into the peritoneal cavity, died, whatever course of after-treatment was pursued; and also that one of the animals, to which nothing was done, lived the longest after the experiment.

There is certainly less chance of being deceived as to the results of experiments, when the poison is introduced either into the cavity of the peritoneum or into the subcutaneous cellular substance than when it has been injected into the stomach, if the animal be allowed to vomit; and I (M. *Orfila*) regret therefore that M. *Rognetta* has not pursued his researches in this respect further.

But there is another consideration which it is most important to bear in mind in reference to the present discussion; and that is, when do we ever resort to bleeding immediately after the swallowing of a poison? On no occasion that I know of; for every one knows that *such a practice would only tend to accelerate absorption.*

Let it be also remembered that in 99 cases out of a 100 arsenic is taken in the solid state, when taken by or administered to any person; whereas it was given to the dogs in a fluid form.

In conclusion, M. *Orfila* stated that, while he did not pretend to affirm that depletory measures were necessary or suited to all cases of arsenical poisoning,

---

* In poisoning from arsenic, M. *Orfila* recommends the use of the hydrated tritoxide of iron. The reader will find an account of the first experiments made with this ferruginous preparation in the Medico-Chirurgical Review for January 1837, and also the history of a case of poisoning alleged to be successfully treated with it, in the number for April 1839.

he was by no means satisfied of the superiority of a tonic mode of treatment by the results of M. *Rognetta's* experiments.*

M. *Dupuys* agreed with most of the remarks of the preceding speaker. The circumstance of the œsophagus not having been tied after the poison was given, and of the arsenic being in the fluid state, rendered the conclusions to be drawn from M. *Rognetta's* experiments of very doubtful authority.

M. *Bouillaud*—as all our readers know, a most ardent disciple of the Broussaian School—remarked, " I can scarcely believe that the fact of vomiting was the only cause of the difference between those animals which were bled or left to themselves, and those which were treated on the tonic and stimulant plan.

I must acknowledge that I was very much surprised at seeing the dogs swallow so greedily, and retain in their stomachs the alcoholic liquids, from which they suffer so much in a state of health. I was, to say the truth, in a new world of unexpected and most strange impressions, which were the more remarkable when I afterwards saw, on the dissection of the animals, the inflamed state of the gastric mucous membrane; while in those, which had been bled, there was scarcely a trace of inflammation. I repeat that it seems to me that the vomitings alone will not account for the successful effect of the stimulant treatment; for I am not aware that the one set of dogs vomited more than the other set which were blooded.

I do not wish to apply the results of M. *Rognetta's* experiments to what takes place in the human body, when arsenic has been taken in a poisonous dose; I confine my remark entirely to what I observed myself in the animals that were the subjects of the experiments; and I must again state it as my opinion that in all probability those dogs, which were treated with stimulants, would have perished more quickly if they had been left to themselves, and nothing had been done to them. It does not follow from these admissions that I reject altogether the employment of bloodletting, in cases of arsenical poisoning. On the contrary, I believe with M. *Orfila* that it is useful, nay necessary, whenever reaction comes on.

This apparent inconsistency in my views will cease when I state it as my opinion that arsenic seems to me to act poisonously on the system in two very different ways; *first*, by exerting a specific influence on the blood, changing its properties, and thus giving rise to *adynamia;* and, *secondly*, by exciting an inflammatory and sthenic action in the parts to which it is directly applied.

M. *Chervin* mentioned that he had assisted at many of the experiments performed by M. *Rognetta*, and that the impression on his mind from what he saw was quite favourable to the employment of stimulants. At the same time he was ready to admit with M. *Bouillaud* that the peritoneal phlegmasia must necessarily complicate the results.

M. *Emery* stated that, at the Hospital St. Louis, where the internal exhibition of arsenic is sometimes pushed to the dose of a quarter of a grain, whenever any unpleasant symptoms arise, they are always of a more or less decided inflammatory character—such as quickened and strong pulse, sharp pains in the region of the heart, general feverish heat, &c. Along with these symptoms he has occasionally observed a partial paralysis of the extensor muscles of the hand. In all such cases he has seen benefit follow the employment of bloodletting.

M. *Amussat* professed himself quite a convert to the views propounded by M. *Rognetta*, and stated that, if he had a patient suffering from the effects of a

---

* At a subsequent period of the *seance*, M. *Orfila* alluded to two periods or stages in poisoning from arsenic; the *first* requiring the use of emetics and chemical antidotes, perhaps also of stimulants, if subsequent experiments confirm the results of those by M. *Rognetta*; in the *second*, bloodletting is *parfaitement indiquée*.

poisonous dose of arsenic, he should certainly give him stimulants, combined perhaps with narcotics.

M. *Renaulden* admitted the utility of general and local bleedings in cases where small doses of arsenic had been taken : but where the dose has been very large, perhaps all treatment is equally fruitless, and bleeding produces no better effect than any other remedy.

M. *Marc* was not ready to adopt the views of the preceding speaker in reference to the extreme rapidity of the danger and the fatal intensity of the symptoms whenever a large dose of the poison has been taken. He remembered having seen a woman who lived for eleven days, (during the whole of which time she was almost incessantly vomiting) after having swallowed an ounce of arsenic and two drachms of corrosive sublimate. Small doses of poison will often cause death much more rapidly than large ones.—*Memoires de l'Academie.*

*Remarks.*—The facts elicited in the discussion, of which we have now given a brief account, are highly interesting, and will suggest some useful hints in a practical point of view. We cannot indeed expect to discover any remedial means which hold out much prospect of constant, or even of very frequent, benefit in poisoning from large doses of arsenic. Not only is the depression of the nervous and circulatory systems often fatally great, but there is at the same time such structural disorganisation of the stomach and bowels induced by the contact of the poison when it has been swallowed, that there cannot be the slightest chance of ultimate recovery. *The action indeed of arsenic seems to be partly irritant and partly narcotic.* It is doubtful whether the latter effects are attributable to a direct influence on the heart, or to a primary influence on the nervous system. In the experiments of *Brodie* and others, it was always found that the action of the heart was excessively feeble, although other muscular parts, including the intestines, nearly retained their usual irritability. The collapse and prostation have in many cases seemed to be much greater when the poison was applied to an external wound than when it had been taken into the stomach.

That arsenic seems however to have an almost specific tendency to irritate and inflame the gastric surface, appears from the well ascertained circumstance, that, in several cases of fatal poisoning from its external application, the mucous membrane of the stomach and bowels has on dissection been found highly vascular and even ulcerated. On the other hand, several fatal cases are recorded where no such appearances have been discovered, even although the poison had been swallowed. On the whole, we may conclude that the salts of this metal are narcotico-acrid poisons—acting as debilitants and sedatives of the nervous and circulatory functions, and as stimulants and violent irritants of the surfaces to which they are directly applied.

The following paragraph from Dr. *Christison's* classical work on poisons furnishes an apt commentary on the whole subject.

"The symptoms of poisoning with arsenic may be advantageously considered under three heads. In *one* set of cases there are signs of violent irritation of the alimentary canal and sometimes of the other mucous membranes also, accompanied with excessive general depression, but not with distinct disorder of the nervous system. When such cases prove fatal, which they generally do, they terminate for the most part in from 24 hours to three days.

In a *second* and very singular set of cases there is little sign of irritation in any part of the alimentary canal ; perhaps trivial vomiting or slight pain in the stomach, sometimes neither ; the patient is chiefly or solely affected with excessive prostration of strength and frequent fainting ; and death is often delayed beyond the fifth or sixth hour.

In a *third* set of cases life is commonly prolonged at least six days, sometimes much longer, or recovery may even take place after a tedious illness ; and the signs of inflammation in the alimentary canal are succeeded or become accompanied about the second or fourth day, or later, by symptoms of irritation in the other mucous passages, and more particularly by symptoms indicating a derangement of the nervous system, such as palsy or epilepsy.—*Rev.*

### Experimental Researches on the Oxydes of Iron as Antidotes of Arsenic.

The readers of the Medico-Chirurgical Review (vide number for January, 1837) are aware that a German chemist, M. *Bunsen,* has proposed the hydrated peroxyde of iron in a moist state as an antidote to arsenical poisoning.

The value of the remedy has been confirmed by several other experimenters; and as the compound formed by the union of the two metallic salts is quite insoluble and inert, there is every reason to believe that, if a complete union could take place in the stomach when the poison has been swallowed and before it has produced its deleterious effects, many a life might be saved. As far as we know, there is only one case on record where the peroxyde has been used in the human subject; and in that case it was used with success. It is reported in the Revue Medicale, and an account of it was given in one of our recent numbers.* The only objection that has been made against the moist peroxyde is the tediousness of preparing it, its inconvenient bulk, and the difficulty of keeping it for a length of time.† A committee was recently appointed by the Society of Medicine to examine the subject in all its details, and to endeavour to discover a substitute for the moist peroxyde recommended by M. *Bunsen.*

The report of this committee has been published, and we shall extract one or two of its most important paragraphs.

After a few prefatory remarks, it is stated, " we do not hesitate to assert that the arsenious acid is *victorieusement combattu,* not only by the moist peroxyde of iron proposed by M. *Bunsen,* but still more effectually and more easily at the same time by the dry hydrated peroxyde—the subcarbonate of iron of the chemists.

This is readily prepared by precipitating the proto-sulphate of the metal by carbonate of potash and washing the precipitate repeatedly. By exposing it freely to the air during desiccation, it becomes completely oxydated, loses its carbonic acid, and is then in short a dry hydrated peroxyde:—100 drachms of this precipitate, when calcined, yield 69 drachms of an anhydrous peroxyde.

The reporters acknowledge that the wet preparation recommended by M. *Bunsen* has in many experiments fully answered the promises held out by that chemist, provided it be administered in sufficiently large doses. But, as we have already said, the objection to this preparation is the length of time required to make it, and its most inconvenient bulk. If therefore the dry peroxyde will answer as well, it must be, considering the facility with which it may be obtained, altogether preferable. Now in numerous experiments performed by the reporters, its antidotal efficacy was very remarkable when the dose of the poison had not exceeded four or six grains.

The first thing to be done is to encourage vomiting as freely as possible. But

* Vide Medico-Chirurgical Review, April 1839.

† The mode of preparing it is by peroxydising a solution of sulphate of iron by the means of nitric acid with the aid of heat, and then adding pure liquor ammoniæ: the supernatant liquor is to be drawn off the flocculent precipitated peroxyde; and fresh water must be poured on it and decanted off several successive times. The process requires, according to M. *Guibourt,* four or five hours.

in doing this, the patient should be made to drink copiously of milk or of oil, and not of water, to prevent the further solution of the poison. Whenever the vomiting is carried as far as is deemed adviseable, a quantity of lukewarm water, in which several ounces of the dry peroxyde of iron are suspended, should be administered. If it is rejected by vomiting, so much the better; we have only to repeat the dose immediately.

The Committee suggest that half an ounce of the dry peroxyde should be given for every grain of the arsenic that may be in the stomach.

For the particulars of the experiments, chemical and physiological* (on dogs), for the purpose of determining the effects of the peroxyde in neutralising arsenious acid, we must refer our readers to the numbers for May and June, 1839, of the—*Revue Medicale.*

---

### ON THE DETECTION OF ARSENIC IN THE BLOOD, FLESH, AND VISCERA, AFTER DEATH FROM POISONING.

M. *Orfila* has, for the last two years, been engaged in a series of experiments to determine whether the presence of arsenic can be detected in the blood and the solid parts of the body after death in cases of fatal poisoning. During last year, he submitted to the attention of the Royal Academy of Medicine a chemical process—which consisted in treating the parts subjected successively with nitrate of potass, sulphuric acid, and with potassa and alcohol—for this purpose. It is however very tedious and also uncertain; and he has therefore substituted another, which he deems to be a great improvement: it is the following.

Take the liver, spleen, lungs, or a certain quantity of the blood of the animal, that has been poisoned with arsenic, and dry them thoroughly. The dried mass is then to be treated in the heat with concentrated and purified nitric acid; in the course of a quarter of an hour or so the whole mass will be dissolved.—The liquor is concentrated; a dark point appears, and soon the carbonisation is complete. It is then to be treated with cold water. The solution is introduced into *Marsh's* apparatus and submitted to the hydrogen test.

In performing the experiments, we must carefully be upon our guard not to raise the temperature of the mixture too high, and also not to use too large a proportion of nitric acid. If more than sufficient be used, the flame is too vehement; and almost all the arsenic will be destroyed. Again, if the heat applied be not sufficient, the carbonisation is not complete, and a disagreeable pyrogenous odour, similar to that of burnt horn, is emitted. Several hours are required for the experiment; and sometimes, even with the greatest care and dexterity in performing it, a portion of the arsenic is lost.

The following are the proportions of nitric acid recommended by M. *Orfila*, to be used with the different parts of the body submitted to the experiment. For three ounces of dried blood, seven ounces of the acid are required; for one ounce of dried gelatine, one ounce of acid; for three ounces of the residue obtained by decoction of the limbs, nine ounces of acid; for six ounces of dried cerebral matter, four ounces of acid; and for all adipose matters, a very large proportional quantity of the acid is necessary. Twelve ounces of dried liver require thirty-four ounces of acid; whereas twenty-two ounces of muscle require only sixteen ounces.

By following the proportions now given, and provided at the same time care

---

* It is mentioned in the report that *small doses of arsenic,* say four or six grains, *proved more quickly fatal than larger ones,* as from 12 to 40 grains. The absorption seems to go on more rapidly when the dose is small.

has been used in effectually drying the substances submitted to experiment, *ou est sur de reussir presque constamment.* We may not indeed succeed in recovering all the poison, but much less will be lost in this than in any other way.

M. *Orfila* gave the particulars of the following instances, in which he had successfully practised the method now described.

1. In a dog, which died after having swallowed 12 grains of arsenic, all the organs were submitted to the experiment; and in every one of them traces of the metal were discovered.

2. The liquid obtained from the decoction of the limbs of *Soufflard,* although previously treated with sulphuric acid, gave the same results.

3. Very recently in the case of a man of the name of *Lorrain,* who died after having taken a large dose of arsenic, a part of his limbs being treated with nitric acid and carbonised, and then submitted to the proper tests, furnished the well known signs of its presence.

4. The same was the case in the liver taken from the body of the man *Mercier.*

5. Lastly, in a woman who poisoned herself a month or two ago, equally satisfactory results were obtained by experimenting on ten ounces of blood which had been taken from the arm. This patient recovered.—*Memoires de l'Acadamie.*

If the expectations of M. *Orfila* be confirmed by the researches of other physicians, the science of toxicology will have achieved a most important discovery. It is well known that if a person, to whom a poison has been given, survives for several days, perhaps no chemical traces whatever of its presence in the stomach or intestines will then be detected on dissection, although the death has been indisputably owing to its effects. The poison has in fact been absorbed and taken into the circulation. That such is the case with arsenic, if the quantity taken has not been excessive, cannot now be questioned for a moment; as M. *Orfila* has succeeded in detecting it not only in the blood, but in almost every one of the solid textures of the body, when all traces of its presence in the intestines had disappeared. The following illustrative case will be read with interest.

On the 22nd of December, 1838, a man died at ——, with all the symptoms of being poisoned. He was however buried, without any examination having taken place.

A fortnight afterwards, the body was disinterred, and submitted to anatomical inspection. The intestines exhibited all the usual lesions characteristic of poisoning by arsenic. Their contents were examined with great care by some experimental chemists; but no traces of arsenic could be detected: the body was again buried. Fresh suspicions however having arisen about three or four months later, M. *Orfila* was then consulted. The body was again taken up and sent to Paris for the purpose of being examined by him. It was in a state of complete decomposition at the time: the stomach and intestines no longer exhibited any traces of their original structure, and all the other parts were more or less disorganised. Notwithstanding the circumstance of the body having been twice exhumed, and submitted to all the mangling of a *post-mortem* examination, not to mention its decomposed state from putrefaction, M. *Orfila* succeeded in detecting the presence of arsenic in the liver and in the limbs, as well as in the water of the barrel in which the rest of the body was kept.

We may also mention that he has obtained unequivocal traces of this metal in the blood, which had been taken from the arm of a patient to whom this poison had been given. It is therefore of great consequence to withdraw a certain portion of blood from the arm in all cases of suspected poisoning.

We must not however forget that, according to some recent analyses of animal bodies, it would seem that there is a minute quantity of an arseniate of lime in the bones, and perhaps also in some of the other textures, in the healthy state. M. *Orfila* admits the truth of this statement himself, but he has pointed out, at the same time, how the fact does not affect the results of medico-legal enquiries.

His experiments clearly show that the natural arsenical compound is not soluble in boiling distilled water, (kept constantly neutralized by an alkali,); whereas, on the contrary, the poison, absorbed into the circulation and disseminated through the organs of the body, is readily extracted by boiling for some hours in water.

We need scarcely say that it is of the utmost importance that the chemist should be most careful as to the purity of all his vessels, whether metallic or earthenware, and of all the chemical tests and agents which he uses, in any medico-legal examination to detect the presence of arsenic in animal bodies. A minute portion of this metal has, for example, been occasionally found in the sulphuric and nitric acids of commerce; also in vessels of iron and tin: and, lastly, in the composition of certain soils.—*Gazette Medicale.*

---

### M. MAGENDIE ON THE ALTERATIONS OF THE BLOOD IN DISEASE.

M. *Magendie,* in his lectures on the Physical Phenomena of Life recently delivered in Paris, has, it may be supposed, carried all his favourite doctrines *a l'outrance.* It is not an easy matter to give the reader an idea of the contents of these lectures, as they embrace so many topics, and are mainly taken up with the record of numerous experiments on living animals—poor brute creation—and of the conclusions which our author has deemed that these experiments warrant.

His great aim seems to be to account for most of the phenomena of life by the same laws which are found to operate on dead or inorganic matter. Thus, for example, the function of absorption—hitherto regarded as a vital act—is attributed altogether to what our athor terms *inhibition,* another phrase for capillary attraction. How he can reconcile this notion with the manifestly *elective* power of the lacteals in absorbing certain matters and rejecting others, does not very clearly appear; but M. *Magendie* has a most convenient way of getting rid of difficulties—he only denies the truth of the objection, and challenges implicit reliance to all his own statements.

As an instance of this very comfortable self-assurance, he takes for granted that his favourite dogma of the veins being the proper, indeed the only, absorbing vessels of the body is assented to and admitted by all physiologists—although there is, perhaps, not a single one in Europe who agrees with him to the full extent on this point. Again, he boldy hazards the assertion that all poisons act *solely* by being absorbed into the current of the circulation, and that the rapidity of their operation is always proportionate to their solubility, and the facility with which they may be absorbed

Such an assertion is, we need scarcely say, quite at variance with the opinions of the most able physiologists as to the *modus operandi* of prussic acid, opium, tobacco, and such like poisons.

The highly important and curious subject of contagion occupies a considerable share of M. *Magendie's* attention.

Here, again, he is at issue with most of his brethren. The French quarantine, it would seem, recognises (we will not say with what propriety) five contagious diseases—plague, typhus, yellow fever, cholera, and leprosy. According to our author, "The contagion of the last four of these diseases is positively denied by every intelligent physician."

It is quite amusing to observe the cool assurance with which this *dictum* is put forth.

To pass to the hitherto obscure subject of the ætiology of typhus and yellow fevers, M. *Magendie* at once solves the difficulty, by informing us that we have only to inject into the circulation of an animal a few drops of putrid animal matter, and then we shall have all the symptoms of these diseases manifested. Hence, says he, we may reasonably conclude that the cause of these fevers must be the absorption into the system of the miasm arising from decayed animal matters. In proof of the truth of this, he alludes to the instance of a vessel laden with fish from Newfoundland, which was stranded on some part of the French coast: the fish putrifying, the neighbouring village became the focus of a pestilential typhus fever; and this continued to rage, until the putrid fish was washed into the sea.

This reasoning will not satisfy the medical logician that there are no other causes of yellow fever, but the miasma of decaying animal matter. M. *Magendie* entirely omits from his consideration the influence of marsh miasmata: but for what reason we do not well know, except that such an admission does not quite accord with his theories.

But, while objecting to the dogmatic tone of the assertions of our author, let it not be supposed that we regard them as altogether visionary and untrue.

It has long appeared to us that the various theories which have been proposed, during the last half century, to account for the phenomena of pestilential fevers cannot stand the test of rigorous examination, and that the leading and essential defect in them all is that the changes in the circulating fluid have been too much overlooked. It has unwisely been attempted to refer every symptom and phenomenon to changes in the solids alone; and thus the practice, which has been based upon this supposition, has been directed almost exclusively to mitigate or remove such changes. The Broussaian school has urged this doctrine to the most *outré* and the most pernicious extreme. A new state of things seems about to succeed.

That a modified and restricted system of humoral pathology has been springing up of late years in France, as well as in our own country, is no doubt well known to all our readers. In the last number of this (the Medico-Chirurgical) Review, we gave a brief notice of a clever paper by M. *Raciborski* on the state of the blood in inflammatory and in typhoid diseases, and we then quoted the opinions of that admirable pathologist, M. *Andral*, in confirmation of the writer's views.

In his recently-delivered lectures on the blood, M. *Magendie* has carried this, as he usually does every other, notion to a most extravagant length, and he treats with the utmost contempt the researches of modern pathology, or pathology of the mere solids, which he designates as a mere chaos and a science which yet remains to be created. He seems to think that no advantage can result from morbid anatomy of the solid parts alone, unless a scrupulous attention be given at the same time to the state of the fluids of the body. In proof of this he appeals to the history of fevers.

The rapidly fatal progress of some he attributes to a sudden alteration in the constitution of the blood itself; and, in this respect, he adopts very nearly the views of the older humoral physicians, *Van Swieten, Huxham*, &c. "For example," says he, "a woman was brought into the Hôtel Dieu with all the premonitory symptoms of some severe acute disease—it was believed to be of variola. In the course of the afternoon, the state of the patient became suddenly much aggravated; *purpura* shewed itself, and death supervened within 30 hours.

Now the phenomena found on dissection in this case were exactly the same with those which I have repeatedly shewn you in the bodies of animals, into whose circulation I had injected some poisonous or putrid fluid.

In such cases, and in similar rapidly fatal diseases, the blood we find has lost its property of coagulating when drawn. Now—what seems rather strange at first—whenever this condition of the blood exists, the circulation appears to be more seriously embarrassed than from any other cause."

A few remarks on this topic may be interesting, and explain the circumstances here alluded to.

When we consider that the blood consists of a fluid which holds in suspension numerous globules of a determinate size and form, and when we compare the size of these globules with the excessively small calibre of the capillary vessels through which they pass, we are surprised that the circulation can go on so evenly and uninterruptedly as it is known to do.

Our surprise must be increased when we find that fluids of much less dense composition than blood, nay that pure water itself, cannot be made, even when considerable force is employed with the injecting syringe, to pass readily from the arterial into the venous set of vessels after death. True, we must bear in mind that the living textures of the body may have properties and have powers of accommodation, which are, in a great measure, lost after life is extinct; but still it will be admitted as a remarkable fact that fluids of thinner consistence than blood should find so much difficulty in passing through the minute vessels.*

The explanation—at least in part—of this circumstance would seem to be that, whenever the tenuity or thinness of the blood is greater than in health, there is a marked tendency to its exuding from the minute vessels, and thus becoming extravasated into the adjoining tissues. But whether this explanation be correct or not, it is quite certain that any change in the composition of the blood is immediately followed by a disturbance of the circulation, and by a series of morbid symptoms which are found to vary according to the nature of the change. If the blood has become thinner, there will inevitably supervene either engorgement or even inflammation of some organ or organs, or œdema, or purpurous patches, &c. Now let us consider what are the conditions of the system, and what are the causes which induce a preternatural thinness of the blood, —premising that the consistence of this fluid depends chiefly on the proportion of the fibrine to the other ingredients.

I will mention to you, says M. *Magendie*, addressing his pupils, an experiment, which I have repeatedly performed:—

If any substance, which will unite and form a compound with the fibrine, be injected into a blood-vessel, the blood will very quickly lose its power of coagulation. Now any of the alkalies will effect this; they combine with the fibrine and form *fibrinates;* and the consequence of this combination is that the fibrine will no longer coagulate, when the blood is drawn and allowed to rest.

This experiment shews that the blood may lose its property of coagulability, without any necessary deficiency in the quantity of fibrine from some abnormal change in its properties. It is, therefore, not to the mere diminution of the quantity of this ingredient, so much as to a change in its properties, that we are to ascribe what has been called a *dissolved* state of the blood. Such a change of the circulating fluid exists in all malignant fevers; and it is owing to the difficulty and embarrassment of transmission which this altered blood must experience—as explained in the preceding page—in the capillary vessels, that œdema, congestion, inflammatory engorgement, hæmorrhages, petechiæ, and other similar symptoms are so frequently observed in these diseases. The pathological

---

* M. *Magendie* says, that, whereas water cannot be made, by any force of impulsion, to pass through glass tubes of less than one-tenth of a *millimetre* in diameter, the diameter of the capillaries does not exceed one-eightieth or even one-hundredth of a *millimetre.* If we inject water into the mesenteric artery of a frog, a very small portion only will reach the mesenteric vein, the greater part having become effused into the surrounding textures. M. *Magendie* suspects that *Ruysch*, the Dutch anatomist, who acquired so high a repute for his minute injections of the blood-vessels, probably made use of some fluid which had some analogy in point of consistence with the blood itself.

appearances found in the lungs, brain, intestines, &c. are all *primarily* attributable to an altered state of the blood; in consequence of which, this fluid has exuded through the capillary vessels in certain parts.

It is therefore of first-rate importance to ascertain the condition of the blood in the early stages of these fevers, as we may be quite assured that their fatality will be always proportionate to the extent or degree of the changes now alluded to.

For this purpose M. *Magendie* has for some time past been much in the habit of employing *saignées exploratives* or very small bleedings, with the view of determining the physical and chemical conditions of the blood, and more especially that of its coagulability. His prognosis of the case is greatly influenced by the results of such an examination.

The causes, which most frequently diminish the coagulability of the blood, are certain conditions of the atmosphere, aerial miasms, certain gases, unnutritious food, alkaline medicines, &c. A dissolved state of the blood is also usually met with in persons who die from lightning, and from the fumes of carbonic acid.

It is from the absorption of the deleterious miasms, which arise from the decay of animal and vegetable substances, into the circulation and from the consequent alteration of the blood, that we are to trace all those pestilential fevers endemic in hot climates, and in the close filthy localities of large towns. The black vomit of the West Indies is well known to proceed from an exudation of the altered blood from the capillaries of the stomach and duodenum. The bloody stools, the petechiæ on the skin, the ecchymosed spots on the lungs, &c. are all very striking proofs of the same morbid state.

We have said that, according to M. *Magendie*, alkaline medicines have a tendency to attenuate the blood, in consequence of their combining with its fibrine and rendering it less viscid.

Acids on the other hand—at least some, and among these the sulphuric is one—are found to increase the viscidity of the blood. Now it would seem—however strange and apparently inconsistent it may strike the mind at first—that similar morbid phenomena are induced by these opposite states of the circulating fluid. Both these states have a tendency to give rise to inflammations and congestive *engouements*. When the blood is too viscid, it cannot permeate the minute vessels: these become obstructed in consequence, and the usual train of symptoms of local inflammation ensue. On the other hand, when the blood is too much attenuated, it exsudes from the capillaries; and thus engorgement and swelling are induced. M. *Magendie* alludes particularly to the tendency to pneumonic congestion, whenever this state of the blood is present: indeed, he carries the dogmata of the humoral pathology even farther than the disciples of Boerhaave and Van Swieten did.

We need scarcely inform our readers that we cannot assent to the opinions of M. *Magendie* on the subject of inflammation. With a few grains of truth there is a bushel of gratuitous assertions. That however he has succeeded in showing that not a few of the phenomena, which have been regarded by so many of his countrymen of late years as infallible signs of existing inflammatory action, are in truth indications of a dissolved and attenuated state of the blood, we are most ready to admit, and to give him praise and credit for.

Let not therefore the mind of the young practitioner be too much pre-occupied with notions of active or sthenic inflammation, whenever he finds, either during life or on dissection, the capillaries of certain parts engorged and distended. Such appearances are, as M. *Magendie* correctly states, often the effects of an attenuated condition of the blood.

If such then be the case, the inference is at once obvious that the detraction of blood and other depletory measures under such circumstances must necessarily aggravate the danger of the patient. No one can doubt that much mischief has been caused by the indiscriminate adoption of such a mode of treatment in all febrile disorders. We cannot enter upon this most important subject at present, and will therefore refer our readers to some valuable practical remarks by M. *Lombard* of Geneva, which will be found in the Foreign Periscope of our last number. We gladly however avail ourselves of the present opportunity of directing the attention of all to an admirable paper on the treatment of typhus fever by stimulants, which appeared from the pen of Dr. Stokes in a recent number of the Dublin Journal.

This accomplished physician has there pointed out with great judgment the aid, which may be derived from auscultation of the heart, in determining the line of practice to be adopted in certain malignant fevers. Whenever its impulse is weaker, and its sounds are fainter and less distinct than in health, we may with confidence, says he, administer stimulants, however much other symptoms may seem to contra-indicate their use.

Dr. *Stokes* strongly recommends that the action of the heart, much more than the state of the arterial pulse, should be carefully and regularly examined in the progress of fevers. The truth of the remark may be extended with great benefit to the therapeutic management of many other diseases. The information which we thus acquire of the energies of the system, is much more exact than can be obtained from the mere examination of the pulse. Dr. *Stokes* has adduced the reports of numerous cases, with the view of showing how the impulse and the sounds of the heart become gradually more and more healthy under the use of large doses of wine and brandy, and how he was thus enabled to guide his practice in cases, where the patient was laying comatose and oppressed with all the symptoms of malignant fever.

In not a few of the cases, upwards of a bottle and a half of wine was administered in the course of four and twenty hours.

---

### ON THE DIAGNOSIS AND TREATMENT OF STOMACH DISORDERS.

The object of the subsequent remarks is to point out the variety of stomach ailments and diseases—very different from each other in their nature and each requiring very different modes of treatment—which have of late years, and more especially among my countrymen, been most erroneously blended together and arranged under the term *gastrite chronique*.

" Every one," says Professor *Debreyne*, " knows that the great multitude, or rather the apparent frequency of chronic gastritis arises, on the one hand, from the extravagant extension of the principles of the self-called *physiological medicine*, and on the other hand, from the prepossession in the minds of many practitioners in favour of the system of universal irritation, and therefore in favour of antiphlogistic remedies in treating them.

All pains and disorders are attributed by these men to chronic irritation or inflammation of the gastric mucous membrane. Whenever the tongue is somewhat redder than usual, and there is any uneasiness in the epigastric region, loss of appetite, and irregularity of bowels, recourse is had at once to leeches, gummy drinks, and a starving regimen.

...... A disciple of the *physiological*, or Broussaian, school sets himself to treat a case of chronic gastritis. He recommends—and very properly—the remedies just now mentioned. Next day the symptoms are relieved; but as there is still some tenderness in the epigastrium, the leeches are ordered to be repeated. Again relief is experienced. The epigastric pain however continues from day to day in spite of the vigorous depletory treatment. The patient too becomes gradually weaker and weaker; his pulse quickens, and a slow fever is lighted up in his system; while all the while the appetite is pretty good, or even craving. We need not pursue such a history, as we shall presently point out how such a case should be treated.

...... A second patient presents himself, labouring under atony of the stomach, which is usually characterised by derangement of the digestion, and uneasiness or even actual pain in the epigastric region.

Now this is often considered a case of chronic gastritis; and it is also treated with leeches, low diet, &c. The immediate result of this treatment is an increase of the general weakness. Perhaps however the epigastric uneasiness is relieved —a most deceptive sign of real amendment, and one likely to mislead the inexperienced practitioner. Probably the application of the leeches is repeated, under the mistaken idea that there is still some lurking inflammatory irritation.

...... Now let us take another case, that of a patient affected with a gastralgia, or gastrodynia. In such cases, along with the severe pain in the region of the stomach, there is usually loss or impairment of appetite, and other symptoms of a disordered digestion.

There can be no mistake here, it will be said by the *physiological physician* that a genuine gastritis exists. Numerous leeches are therefore applied, and a most strict *diete* is enjoined.

Now almost every pain—whether phlogistic, rheumatic, neuralgic, or even atonic—is relieved for a time by the application of leeches in the neighbourhood of the affected part. In neuralgic and atonic pains, the relief is however delusive and only temporary; for next day, or even sooner, they are as severe as ever. If this mode of treatment be therefore continued, the patient's strength is more and more exhausted, and the disease probably aggravated at the same time.

...... Then again consider the symptoms of a patient affected with scirrhus or incipient cancer of the stomach. He suffers from a sense of continual weight, and from a dull and deep-seated pain, especially when the stomach is empty, from most troublesome flatulence, acidity, frequent vomiting of a glairy matter, constipation, &c. The antiphlogistic treatment—under the impression that the case is one of chronic gastritis—is followed by nearly the same effects as, we have pointed out, are apt to follow in atony of the stomach.

...... Lastly, in cases of ordinary *embarras gastrique*—characterised by loss of appetite, a bitter taste in the mouth, a coated tongue, nausea, vomiting of bilious matters, sensibility of the epigastrium, headache, &c.—the physiological physician has often been sadly in error in supposing that the antiphlogistic treatment was at all necessary for its relief......

Dr. *Debreyne* then details with great minuteness the chief diagnostic symptoms of each separate case.

In treating of *genuine* chronic gastritis, he dwells particularly on the assistance which may be derived, in discriminating the disease, from watching the effects of different sorts of food upon the severity of the symptoms. 'Whenever,' says he, 'a farinaceous and milk diet is well borne, or at least is better borne, and causes less uneasiness than any form of animal food, even that of simple broth, we have strong reason to suspect the presence of some degree of inflammation. This symptom may be considered as a sort of touch-stone, by means of which we may in general distinguish the irritative from the purely nervous and atonic affections of the stomach. True, it is not an infallible indication; but it is certainly one of the greatest value to assist our correct diagnosis. Of this we may be assured, that whenever an animal diet suits a patient better than a vegetable one, the case is not one of gastritis.'

Now for a word or two respecting the treatment of *genuine* chronic gastritis.

As a matter of course, local detraction of blood, spare diet, refrigerants &c. are to be used. But if after a due employment of these means, the pain of the stomach still continues, we are not to persevere in the same practice indiscriminately. The pain may now be rather of a nervous than of an inflammatory character; and, if there be good reason to suspect this, a mild opiate in a mucilaginous vehicle will be far more serviceable than further bleedings.

Opium is however, on the whole, much less efficacious in gastritis than in enteritis or in dysentery. We should not persevere in its use, if the pain does not yield in the course of two or three days. Under such circumstances, let a blister be applied upon the epigastrium. Should the pain resist this as well as the opiates, we may then regard it as of an atonic character. Attention to the effects of food, animal and vegetable, will assist our diagnosis in this respect, as already explained.

Small doses of rhubarb or calumba, either alone or conjoined with sedatives, such as hydrocyanic acid, minute doses of morphia should now be tried. A low feverish state is apt to follow a too-long protracted use of depletory and debilitant remedies.

*Atony of the Stomach.*—This disease is of not unfrequent occurrence, especially in women affected with leucorrhœa, chlorosis, anæmia, &c.

It is unnecessary to particularise its symptoms, as all the phenomena well known under the too-comprehensive term 'dyspepsia' may be present. Even pain, aggravated too on pressure, in the epigastrium, is not uncommon. One important character is, that the distress of the stomach is usually increased by the use of vegetable and farinaceous food, and relieved, more or less, by a generous diet of animal meat, wine, &c.

Neither opiates nor antiphlogistics are suited to such a case: tonics alone are the proper remedies.

There may indeed be a certain degree of inflammatory or of neuralgic pain blended, so to speak, with the atonic pain, which is the prevailing element in the cases now under consideration; and then indeed it will be necessary to modify our treatment, combining the use of occasional leechings and anodynes with the tonic plan. An animal diet of the lighter and white meats at first and subsequently of the stronger ones, the use of some mild ferruginous preparation, of quinine, or some other vegetable bitter, such as gentian, calumba, aloes, &c. in the form either of infusion or of wine, constitute the most approved practice.

In a variety of gastro-atony—characterised by frequent returns of vomiting—there is no remedy more useful than calumba in the form of powder, five to ten grains taken three or four times a day. If the epigastric uneasiness should be troublesome, a mild opiate preparation may be mixed with it.

The patient should be advised to take but little of any watery drinks, such as tea, gruel, &c. Seltzer water is a good beverage.

*Gastrodynia and Gastralgia.*—The first of these terms should be applied to such pains in the stomach as are of a rheumatic, and the second to such as are of a purely neuralgic nature. The pain in both is almost always much more severe than in genuine chronic gastritis; and yet it is usually not increased, but is often relieved, by pressure on the epigastrium. Moreover, there is no feeling of heat, nor is there much thirst, and the pulse is not quickened. Generally there is but little derangement of the digestion; and almost all kinds of food may be taken, without any decided effects either as to encreasing or mitigating the pain. In genuine gastrodynia we can usually discover that there has been the retrocession or transport of a previous rheumatic affection of some of the joints, &c. Hence a certain degree of phlogistic action may be coexistent with the pain;—not so however with *gastralgia*.

As to the treatment, opiates are on the whole the most efficacious remedies. In many cases they require to be administered in large doses. Some of the diffusible antispasmodics, such as æther, camphor, the liquor Hoffmanni, &c. are often useful. The sub-nitrate of Bismuth is an admirable preparation in numerous cases. Sometimes, more especially where the disease is of a rheumatic character, the use of blisters, applied either to the epigastrium or to one of the lower limbs, is necessary.

We shall close these remarks with a few words as to the use of opium in painful affections of all sorts of the digestive canal. There is no remedy so generally beneficial, nay, even necessary, as opium in such affections; as, for example, in cramps, colicky pains, dysentery, as well as in gastralgia. In some cases, it must be associated with bloodletting; in others, with laxatives and emetics; and in a third set, with bitters and tonics. Medical men have been, especially during the last thirty years, far too timid in the employment of this admirable remedy in the treatment of disease. They forget that often, very often, Nature only requires to be freed from present pain, to enable her to restore a disordered function to a healthy condition, and a suffering organ to the regular performance of its duties without distress or inconvenience.—*Revue Medicale.*

---

### M. Ricord on the Treatment of the various Stages of Syphilis.

In the Foreign Periscope of the last number of this (the Medico-Chirurgical) Review, there is a brief paper on the views which M. *Ricord*, the experienced surgeon of the Hôpital des Veneriens at Paris, entertains on some of the most important topics connected with the history and treatment of syphilitic diseases. He has adopted a new arrangement of their phenomena, by dividing them into three, in place of two—as hitherto—groupes or classes; viz. the *primary*, the *secondary*, and the *tertiary*. The following observations are extracted from a paper which M. *Ricord* himself has recently published in the Bulletin de Therapeutique.

"One of the reasons, which has given rise to so much confusion and misunderstanding as to the proper nature of venereal diseases, is the vain attempt of seeking in one single and identical cause, to which they might all be referred, effects which are uniform and constant, and such as might be remediable by the same therapeutic means.

If it had been only once admitted that in many *reputed* venereal affections, there are some which are quite foreign to, and independent of, the primary and genuine disease, no difficulty would have been experienced in arriving at a more correct and more successful diagnosis and practice.

Besides this important consideration, there is another of equal importance, both in a diagnostic and in a therapeutic point of view; and it is this. Even in genuine syphilitic affections, the phenomena are by no means always the same. Much depends on the duration of the disease, on the tissues affected, on the constitution or idiosyncrasy of the patient, on the diseases which he has had before, &c. &c. Now all these varying conditions require corresponding modifications in the treatment.

By far the best arrangement of syphilitic affections is into *primary, secondary,* and *tertiary*. The *first* includes only those phenomena or symptoms which are produced by the direct local effect of the venereal poison. In the *second* stage or phasis, the morbid principle taken up by the absorbents becomes the cause of a general or constitutional poisoning, the effects of which are multiplied and various. These effects are twofold. In some *secondary* affections, the poison still retains its primary and peculiar characters, and is capable of communicating the disease by inoculation or by direct contact; while in others, this property of contagiousness no longer exists, but another characteristic property—that of communicability from parent to offspring—has been acquired.

In the *third* phasis or stage, the disease has lost alike its contagiousness and its communicability by descent: if it still retains a certain morbid influence on generation, this influence is manifested in the production of diseases, which are foreign to syphilis; for example, to scrofulous affections.

In a patient who has caught the primary disease, and in whom its characters have not been modified by any treatment whatever, the secondary phenomena usually shew themselves in from four to eight weeks. They may indeed appear earlier or somewhat later; but the usual period of their manifestation is as we have now stated. On the other hand, the tertiary phenomena very rarely develope themselves before the sixth month, and in most cases not till after one or even two years, posterior to the original infection.

It has been alleged that the phenomena, which we group under the class of tertiary, sometimes appear without having been preceded by those of the secondary stage. Such an occurrence does not tally with the results of our own experience. The secondary symptoms indeed are sometimes not well marked, and continue but for a short time; or perhaps in such cases they may have been arrested by a judicious regimen.

Let it not be supposed that there is any stated time that the primary, secondary, and tertiary phenomena of the syphilitic disease can be said to continue; for it is well known that the primary may last for even a twelve-month, and the secondary, under various modifications, for two or three years or even longer. All that we wish to inculcate is, that they invariably follow or succeed to each other in the manner that we have stated:—viz. that the primary symptoms invariably precede the secondary, and that the secondary invariably precede the tertiary. They are never observed to appear in an inverse series.

Now as to the seat of, or the tissues affected by, the syphilitic disease in its different stages: The *primary* disease attacks the skin, the mucous textures, the lymphatic vessels proceeding from these different tissues, and lastly the superficial absorbent glands.

The phenomena of the *secondary* stage are observed in the skin and in the mucous tissues both internal and external: of the former, the mucous lining of the throat and that of the extremity of the rectum are most frequently affected. In addition to these, the lymphatic system, the testicles, the iris, the hair and the nails, often suffer from secondary syphilitic disease. When these last-named parts become involved, the disease indeed is verging to the limits of the *tertiary* stage or phasis, during which the deep-seated parts, such as the sub-cutaneous and sub-mucous textures, the bones, ligaments, &c. are chiefly affected.

A fact worthy of notice is that, in the same manner as the primary phenomena may remain altogether local, so the secondary and the tertiary phenomena sometimes cease by becoming local also, and continue in this state, long after the system has been quite freed from the cause which gave rise to them. Of this nature are certain *inodular* indurations remaining in the seat of primary chancres; such also are a great number of excrescences or vegetations about the labia and penis, and various affections of the periosteum and even of the bones.

We shall now proceed to point out the practical value of the three-fold division of syphilitic phenomena which we have laid down, and endeavour to shew how attention to it will greatly influence the success of our treatment.

In the *primary* phenomena, which exhibit no indication of constitutional poisoning by any hardening of the tissues where the ulcers are seated, the employment of mercurial remedies is oftener pernicious than useful. As soon however as any induration around the primary sore takes place—an incontestible proof that absorption either has, or is about to take place—mercury, if it be not absolutely a specific, is at least the most efficacious of all remedies against the disease. It is equally useful in the greater number of cases of *secondary* syphilis, unless indeed the disease be complicated with certain morbid states of the system which contra-indicate its employment.

But very different are its effects in cases of genuine *tertiary* syphilitic disease.

For then it is usually not only not an efficacious remedy, but it is often such a pernicious poison that we cannot wonder that so many writers have not hesitated to ascribe to its use all the worst and most protracted features of constitutional syphilis.

Let it however be well remembered that if the tertiary symptoms are accompanied (as is not unfrequently the case) with any secondary symptoms, then indeed a cautious use of mercury will generally facilitate and expedite the cure. It is only when they (the tertiary) exist alone, that I condemn all preparations of this metal as useless, if not pernicious. The remedy, which I have found of the highest value in such cases, is the hydriodate of potass—a medicine which has been much vaunted, especially in England, as an anti-syphilitic remedy, but which has hitherto been far too indiscriminately used. The formula which M. *Ricord* recommends is a solution of ten grains of the salt in three ounces of water, and one ounce of syrup of poppies—a third part to be taken thrice daily in a decoction of sarsaparilla, hops, or saponaria. The dose should be increased by ten grains or so every five days, so that at length 100 grains are taken in the course of the twenty hours. Its use should be persevered in for a considerable time—say from six weeks to three months.

In some patients the hydriodate causes distress and even acute pain in the stomach; occasionally too vomiting, thirst, and diarrhœa. The appetite is usually increased: so likewise is the action of the kidneys, and hence occasionally a degree of diabetes is induced. The iodine may be discovered in the urine by the appropriate chemical tests. Some patients experience what has been called an *iodic intoxication,* characterised by an unsteadiness in the movements of the voluntary muscles, by slight startings and subsultus tendinum, by heaviness of the head, and a certain degree of confusion of the mental powers. Whenever such symptoms appear, the dose of the salt should be diminished, or its use suspended for a time.

The good effects of the hydriodate, when judiciously and discriminately administered, are usually obvious in the course of the third week or so: tubercles begin to shrink and be absorbed, ulcerations become cleaner and healthier, suppuration diminishes, pains in the bones are less severe, and swellings of the periosteum become less and less. The employment of local means, various according to the circumstances of each case, is not to be neglected at the same time. The application of a wash or gargle, containing iodine in solution, to ulcerations will be found of great benefit.* For pains in the bones, a succession of blisters on the affected part will give most relief. The same remedy will often be found to assist very materially in the dispersion of indolent subcutaneous tumors. In some cases, it seems decidedly useful to dress the blistered surface with mercurial ointment. When tubercular and periostitic swellings are unusually obstinate, the application of the tincture of iodine, pure or diluted with only one or two parts of water, so as to act as a mild caustic, is often beneficial.

I have already said that, if any secondary symptoms co-exist with the tertiary, it will be necessary, or at least useful, to combine the use of some mercurial preparation with that of the hydriodate. According to my experience, no preparation is preferable to the proto-ioduret of mercury, in doses of from one to five grains in the twenty-four hours.

According to the nature of the symptoms which first disappear, so should the continuance or the suspension of the particular remedy be regulated; but, as a general remark it may be stated, that it is well to continue the treatment for two,

---

* M. *Ricord's* usual formula is one drachm of Tincture of Iodine (Pharm. Paris) to eight ounces of distilled water. This wash, when used as a gargle, causes a slight degree of a burning sensation in the mouth: the dose of the tincture requires, in some cases, to be raised to four or even six drachms.

three, or four weeks after the apparent cure of the symptoms for which the remedy was given.

M. *Ricord* closes his observations by alluding to his extended field of experience at the Hôpital des Veneriens, where he has tested on a large scale the value of the practice which he has now recommended.—*Bulletin General de Therapeutique.*

---

### RHEUMATISM OF THE UTERUS.

In the last Number but one of the Medico-Chirurgical Review we called the attention of our readers to what the German and French physicians have denominated by the term, Rheumatism of the uterus,—remarking at the time that, as this organ is truly of a muscular structure, it is highly probable that it may be the seat of rheumatic disease.

The following cases, extracted from Rodamel's *Traité du Rhumatisme Chemique*, exhibit the sort of cases to which allusion is made.

*Case* 1.—A woman, who had for several years been subject to rheumatic attacks, experienced an attack last January in consequence of exposure to cold and wet. The inferior extremities were the parts chiefly affected. Suddenly the pain quitted the right leg, where it had been for several days extremely severe, and fixed itself in the region of the uterus.

There it was very acute and lancinating; abating at one moment and soon returning with great severity. The hypogastrium was tender on pressure, and the uterus felt, on examination, heavier than usual. By the use of remedies calculated to dislodge the rheumatism and to soothe the local irritation, the symptoms were speedily relieved; but no sooner was this relief experienced than the pain returned with force in the left knee.

*Case* 2.—A lady, after her second confinement, became affected with chronic rheumatism in the right thigh. The pain moved about to different parts of the body, and among the rest to the uterine region, giving rise to a sense of constant weight and dull pain there. After some time, it seized the stomach; then moved downwards to the left knee, and again returned to the uterus. The patient had become alarmed that a cancerous disease was beginning to establish itself; but her fears were quickly dissipated by being relieved from her suffering. For several years however she was subject to occasional returns of uterine distress, whenever there was a rheumatic diathesis, so to speak, present in her system.

*Case* 3.—A lady, 63 years of age, suffered in 1820 from flying rheumatism, which successively attacked the left hand, the legs, the right hand, and the legs again. During a subsequent year, the pain fixed itself in the loins, then between the shoulders; again in the loins, and at length in the region of the uterus, where it was of a sharp lancinating character. Every now and then the patient felt as if she had the forcing, bearing-down pains of labour. There was also some degree of tenesmus and of incontinence of urine. By employing means to recall the rheumatism to the lower extremities, all these unpleasant symptoms were speedily dissipated. This patient experienced subsequently several similar attacks.—*L'Experience.*

---

### Utility of Chloruretted Injections into Large Abscesses.

M. *Payan*, surgeon of the hospital at Aix, has reported several cases of the good effects of injecting the chloruret of soda—one part of Labarraque's liquid chloruret mixed with eighteen or twenty parts of tepid water—into the sacs of large abscesses, when their discharge has become acrid and unhealthy, and symptoms of constitutional irritation have come on. It is quite unnecessary to detail the particulars of these cases. Suffice it to say, that M. *Payan* is of opinion that the accession of hectic fever after the opening of large collections of purulent matter is attributable to the fermentation induced by the admission of atmospheric air, and to the consequent irritation of the lining membrane of the sac.

If this opinion be correct, the primary object is to prevent or to counteract the degeneration of the purulent matter; and according to our author's experience, no remedy is so effectual for this purpose as the chloruret of soda.—*Revue Medicale.*

---

### Poisoning from the Extract of Aconite.

The following observations merit the attention of every medical man in a practical point of view, as they shew the necessity of great caution in the administration of certain potent drugs, and more especially of artificial preparations of these, which may vary much in strength and activity. Here we have the account of the deaths of two persons, and of the serious illness of several others, in consequence of their having been taking an alcoholic extract of the Aconitum napellus, for the cure of rheumatic pains, of much stronger powers than that which had been previously administered, although the doses had not been increased in quantity.

*Case* 1.—A man, 45 years of age, had been afflicted with rheumatic pains for upwards of nine months, when he went on the 19th of December into the hospital at Bourdeaux. He was ordered to take two grains of the extract of aconite morning and evening; and under this treatment, the dose having been increased to ten grains daily, the patient nearly got quite rid of his sufferings. On the 12th of January, the patient took his accustomed quantity of the medicine, and within a quarter of an hour afterwards he experienced, as he had been accustomed to do, tremblings in the muscles of the thighs and arms; these, which were somewhat painful, instead of gradually ceasing in the course of a few minutes, became more and more violent, and were succeeded by genuine convulsive movements which lasted for a quarter of an hour or so. During these convulsions the patient lost all sense of consciousness; as they ceased, he answered questions distinctly, but his vision remained confused. He complained of a fixed burning headache, which he said felt as if a bar of hot iron was passed through his brain from one temple to another. He complained of extreme heat in his mouth and throat; and the stomach was so irritable that it almost immediately rejected any food or drink that was taken. The respiration was hurried and uneasy; the pulse slow, irregular, and weak, and the limbs were of an icy coldness. On applying the ear over the cardiac region, the movements and sounds of the heart were heard to be confused and disordered. The physician in attendance, justly regarding the alarming symptoms as all attributable to the narcotic effect of the poison on the nervous system, and more especially on the nerves of the respiratory and circulatory apparatus, as he had seen another patient under similar circumstances sink, on the preceding evening, under the gradual and progressive cessation of the breathing and pulse, immediately administered to the patient a strong infusion of *huaco*—a plant which seems to have a special stimulating effect on the heart—along with repeated doses of ammonia, the application of heat to the extremities, frictions along the spine and over the stomach with tincture of cantharides, &c. By a continuance of these means, the unpleasant symptoms gradually ceased, and the patient recovered not only from the immediate effects of the poisoning, but also from his former rheumatic sufferings.

On the same day, other three patients, to whom the fresh-prepared extract had been given, were similarly affected as in the preceding case. One, who had taken four grains in the morning, died at the end of three hours.

The following is the report of the other case which proved fatal.

A middle-aged man had been under treatment for some time with the extract of aconite for old rheumatic pains and had derived decided benefit, when one morning, almost immediately after taking his dose, which was from some fresh-prepared quantity of the extract, he was suddenly seized with a sense of burning in the throat, repeated vomitings, great anxiety, icy coldness of the limbs and body, syncope, and a sort of stoppage (arret) of the circulation: these symptoms proved fatal in the course of four hours. On dissection, the veins of the cerebral membranes were highly congested, and the substance of the brain, when divided, exhibited numerous points of dark-coloured blood. The lungs also were congested with black blood, and were much less crepitant under pressure than usual. The right cavities of the heart were filled with a thick blood, which had all the appearance of currant-jelly. The stomach was *zebré*, and exhibited on its surface numerous patches of a brown colour, especially towards its cardiac extremity. The liver and spleen were gorged with black blood.

Two other patients in the hospital were affected in a similar manner, but in a less severe degree, as the patient whose case we have briefly reported. By the use of strong coffee, and the application of outward warmth, the symptoms of poisoning gradually subsided and disappeared.

The facts now detailed sufficiently point out the necessity of the greatest caution in the administration of poisonous vegetable extracts from different parcels or quantities prepared at different times, and perhaps under different circumstances. The physician will act wisely to diminish the dose of such drugs, whenever a fresh parcel or quantity is used for the first time.—*Gazette Medicale.*

